# Synergistic anti-cancer activity of *Artemisia vulgaris L.* aqueous extract with cisplatin against A549 lung cancer cell line

**DOI:** 10.1186/s12906-026-05481-5

**Published:** 2026-08-11

**Authors:** Seham Salah El-Din Elhawary, Hadeel Nabil Ahmed, Mouchira A. Choucry, Rasha M. Allam, Osama G. Mohamed, Kamel Mahmoud, Amira K. Elmotayam

**Affiliations:** 1 Department of Pharmacognosy, Faculty of Pharmacy, Cairo University, Kasr El-Aini Street, Cairo, 11562 Egypt; 2 Department of Pharmacognosy, Faculty of Pharmacy and Drug Technology, Egyptian Chinese University, Cairo, 11786 Egypt; 3 Pharmacology Department, Medical Research Institute, National Research Center, 33 El-Bohouth St., Dokki, P.O.12622, Cairo, Egypt; 4 Natural Products Discovery Core, Life Sciences Institute, University of Michigan, Ann Arbor, MI 48109 USA; 5 Department of Pharmacognosy, Faculty of Pharmaceutical Sciences & Drug Manufacturing, Misr University for Science and Technology, Giza, Egypt

**Keywords:** Lung cancer, A549 cells, *Artemisia vulgaris* L., Cisplatin, Autophagy, Apoptosis

## Abstract

**Background:**

The increasing resistance and adverse effects associated with conventional chemotherapeutic agents such as cisplatin have prompted the investigation of natural compounds that act synergistically with chemotherapy to combat cancer. This study investigates the cytotoxic effects of an aqueous extract of *Artemisia vulgaris* L. in conjunction with cisplatin on A549 human lung cancer cells. The research analyzes the phytochemical composition of the extract and its potential mechanisms of action by assessing apoptosis and autophagy. Cytotoxicity studies demonstrated a synergistic interaction between *A. vulgaris* and cisplatin. We utilized molecular docking to examine the interactions of the extract components with critical proteins involved in these processes.

**Methods:**

Liquid chromatography-tandem mass spectrometry (LC–MS/MS) was employed to characterize the phytochemicals in *A. vulgaris*. A549 cells were subjected to various concentrations of *A. vulgaris* L. aqueous extract, cisplatin, and their combination. We utilized the SRB assay to assess cell viability and the combination index (CI) analysis to evaluate the synergistic interaction between the two compounds. Molecular docking was performed for selected compounds against apoptotic and autophagic target proteins.

**Results:**

The combination treatment showed a greater cytotoxic effect than either treatment alone, suggesting a synergistic improvement of anticancer action through the induction of both apoptosis and autophagy. This was manifested by a significant decrease in the IC_50_ values of *A. vulgaris* and cisplatin to 13.32 ± 1.66 μg/mL and 0.44 ± 0.02 μM, respectively, with a combination index (CI) of 0.7. LC–MS/MS profiling showed a wide array of bioactive compounds such as flavonoids, terpenoids, coumarins, and phenolic acids many of which have been found to possess anticancer effects.

**Conclusion:**

The results indicated that *A. vulgaris* L. may potentiate the anticancer effect of cisplatin, potentially allowing dose reduction and minimizing side effects.

**Supplementary Information:**

The online version contains supplementary material available at 10.1186/s12906-026-05481-5.

## Background

Lung cancer represents a major public health concern, being the second most commonly diagnosed cancer and the leading cause of cancer-related mortality globally, surpassing all other cancer types. In 2022, there were 2.48 million new cases and 1.8 million deaths due to lung cancer [1]. According to the age-standardized incidence rate, the incidence of lung cancer in men is twice that in women, and accordingly, the mortality rate in men is also approximately twice that in women. According to global incidence and mortality estimates by region and country, Eastern Asia exhibits the highest incidence rate, whereas Niger in Africa has the lowest, while Egypt has an incidence rate ranging from 7 to 10.9 per 100,000 persons. Annual lung cancer cases are predicted to reach 4.62 million, with 3.55 million fatalities by 2050 [1].

Chemotherapy, especially with cisplatin, remains a cornerstone of lung cancer treatment, but is typically limited by severe adverse effects. Natural products have been found as potential adjuvants to improve the efficacy of cisplatin and, at the same time, diminish its toxicity. Moreover, it has been shown that *Artemisia* species improve the susceptibility of tumor cells to cisplatin, thereby decreasing the therapeutic dose, and *A. vulgaris* showed hepatoprotective activity against cisplatin-induced toxicity in experimental animals [2, 3]. In addition, plant-derived substances like artesunate have been employed to augment the anticancer activity of cisplatin against A549 lung cancer cells, indicating the potential of medicinal plants as supplementary agents in the treatment of lung cancer [4, 5].

*Artemisia vulgaris* L., which is known as mugwort, wormwood, felon herb, moxa, and sailor’s tobacco in English and “Sheeh” in Arabic [6], belongs to the family Asteraceae [7–9], and is extensively spread in Africa, Asia, Europe, and the Americas [10]. It is also known as the “mother of herbs” and has a long history of use in ancient Egypt, Rome, and Greece to heal numerous diseases. The traditional use of *A. vulgaris* has included the management of gastrointestinal, gynecological, hepatic, and neurological illnesses, as well as wound healing and the therapy of inflammatory and infectious diseases. It is also claimed to have anti-inflammatory, antibacterial, antimalarial, bronchodilatory, and antioxidant properties [9–11].

*A. vulgaris* L. has demonstrated antioxidant activity, and its anticancer effect has been established in numerous research studies*. A. vulgaris* L. exhibits cytotoxic action against SW-480 and HCT-15 human colon cancer cells [12, 13]. The essential oil of *A. vulgaris* L. induced apoptosis in HL-60 cells via caspase-dependent pathways [9]. The essential oil nanoemulsions demonstrated antiangiogenic action and alcoholic extracts showed cytotoxic activity against several cancer cell lines including HepG-2, MCF-7, HeLa, and A549 cells [14–16]. Furthermore, it has been revealed that eupafolin and naringin are bioactive ingredients responsible for its anticancer action and the ethanol leaf extract has exhibited antiproliferative and pro-apoptotic properties against A549 lung cancer cells [17–19]*.* Several research studies have been conducted on the anticancer potential of *Artemisia* species in combination with cisplatin, and they have shown increased apoptosis in lung cancer cells [2]. Moreover, the proapoptotic effect of *A. vulgaris* on A549 lung cancer cells has already been described in previous investigations; however, the combination with cisplatin was not evaluated in these studies [19]. To the best of our knowledge, the present work is the first to examine the combined effect of *A. vulgaris* extract and cisplatin on A549 lung cancer cells. The results showed that the combination improved the anticancer effect compared to the individual treatments, which might be attributed to the greater induction of apoptosis and the involvement of the modulation of apoptotic signaling pathways.

## Methods

### Plant material

*A. vulgaris* L. was purchased from the Harraz local market and identified by Dr. Ibrahim Elgarf, Professor of Plant Taxonomy, Department of Botany and Microbiology, Faculty of Science, Cairo University, Giza, Egypt. A voucher specimen (No. 6–7–25-F) was deposited in the Herbarium of the Faculty of Pharmacy, Cairo University, Cairo, Egypt.

### Extract preparation

Twenty grams of dried *A. vulgaris* L. powder were boiled with 100 mL of distilled water at 100 °C three times. The resulting mixture was filtered, concentrated using a rotary evaporator, and finally lyophilized.

### HPLC quantitative analysis

The chlorogenic acid standard was purchased from Sigma-Aldrich Chemical Co. Ltd. (Sigma-Aldrich, Saint Louis, MO, USA). All other reagents, such as trifluoracetic acid, acetonitrile, and methanol, were of the highest analytical grade.

#### Standard preparation

10 mg of chlorogenic acid was used as a standard in 10 mL of methanol and water (50:50) to give a concentration of 1 mg/mL, serial dilutions were then prepared to obtain concentrations of 20, 30, 40, 50, 60, 70, 80, and 90 μg/mL. The solutions were then filtered using a 0.22 μm syringe filter, and 10 μL was injected.

#### Sample preparation

Fifty mg/mL of the prepared extract was dissolved in 1 mL of methanol and water (50:50), sonicated for 20 min, and then filtered using a 0.22 µm syringe filter.

#### Instrumentation and HPLC chromatographic conditions

An in-house method was used for chlorogenic acid as a standard in HPLC analysis [20] with some modifications. The analysis was carried out on a Waters 2690 Alliance HPLC system equipped with a Waters 996 photodiode array detector. Samples were injected onto a Luna cyano 4.6 × 250 mm, 5 μm column. The mobile phase was 0.1% trifluoroacetic acid (A): acetonitrile (B) at the following gradient elution mode: 95% A: 5% B at 0–4 min, 92% A: 8% B at 4–5 min, 50% A: 50% B at 5–7 min, and 95% A: 5% B at 8–11 min, with a flow rate of 1.5 mL/min at ambient temperature and at a wavelength of 324 nm. Samples were run for 12 min.

The limits of detection (LOD) and quantification (LOQ) were calculated according to ICH guidelines using the equations:

$$LOD = \frac{3.3\upsigma }{\mathrm{S}}$$

$$LOQ = \frac{10\sigma }{\mathrm{S}}$$

where σ is the residual standard deviation of the calibration curve regression and S is the slope of the calibration curve.

### Determination of total flavonoids and total phenolic content

Total flavonoid content (TFC) was estimated by the aluminum chloride colorimetric approach of [21] with some adjustments for microplate analysis. A stock solution of quercetin was produced in methanol (1 mg/mL), and twelve standard concentrations were serially diluted. The absorbance values were averaged over six replicates, and a calibration curve was prepared using quercetin as a reference standard (Fig. S1 in the supplementary file).

The aqueous extract of *A. vulgaris* was produced at a concentration of 12 mg/mL in distilled water. 15 μL of sample or reference solution was added to a 96-well microplate. 175 μL of methanol, 30 μL of 1.25% AlCl₃, and 30 μL of 0.125 M sodium acetate (C₂H₃NaO₂) were added. The yellow complex was measured in a FluoStar Omega microplate reader at 420 nm after incubation for 5 min at room temperature. The data were expressed as mean ± SD and as quercetin equivalents (QEs).

Total phenolic content (TPC) was evaluated by the Folin-Ciocalteu method [22]. A stock solution of gallic acid (1 mg/mL in methanol) was produced and serially diluted to yield eight standard concentrations. The calibration curve of gallic acid (Fig. S2 in the supplementary file) was prepared using the mean absorbance of six replicates.

An aqueous extract of *A. vulgaris* was prepared in distilled water at a final concentration of 2 mg/mL. The assay was carried out in a 96-well microplate by adding 10 μL of sample or standard solution to 100 μL of diluted Folin-Ciocalteu reagent (1:10, v/v). Thereafter, 80 μL of 1 M Na₂CO₃ was added and incubated in the dark at room temperature (25 °C) for 20 min. The absorbance of the blue-colored complex was measured at 630 nm using a microplate reader (FluoStar Omega). Results were given as mean ± SD and gallic acid equivalent (GAE).

### UHPLC-QTOF-MS/MS qualitative analysis

Ultra-high-performance liquid chromatograms (UHPLC) were acquired using an Agilent LC–MS system, which consists of an Agilent 1290 Infinity II UHPLC linked to an Agilent 6545 ESI-Q-TOF–MS in both negative and positive ionization modes, with aliquots (1 µL) of the EtOAc extract. Samples (1 mg/mL in MeOH) were analyzed using a Kinetics phenyl-hexyl (1.7 μm, 2.1 × 50 mm) column, employing a 1-min isocratic elution of 90% A (A: 100% H_2_O + 0.1% formic acid), followed by a 6-min linear gradient elution to 100% B (95% MeCN + 5% H_2_O + 0.1% formic acid) at a flow rate of 0.4 mL/min. ESI parameters were established at a capillary temperature of 320 °C, a source voltage of 3.5 kV, and a sheath gas flow rate of 11 L/min. Ions were identified in the comprehensive scan at an intensity exceeding 1000 counts at a rate of 6 scans per second, with an isolation width of 1.3 m/z, a maximum of 9 selected precursors per cycle, and a ramped collision energy (5 m/z/100 + 10 eV). The purine C_5_H_4_N_4_ [M + H]^+^ ion (m/z 121.050873) and hexakis (1H,1H,3H-tetrafluoropropoxy)-phosphazene C_18_H_18_F_24_N_3_O_6_P_3_ [M + H]^+^ ion (m/z 922.009798) served as internal lock masses for positive mode, while TFA C_2_HF_3_O_2_ [M—H]^−^ ion (m/z 112.985587) and hexakis (1H,1H,3H-tetrafluoropropoxy)-phosphazene C_18_H_18_F_24_N_3_O_6_P_3_ [M + TFA—H]^−^ ion (m/z 1033.988109) were utilized as internal lock masses for negative mode. The obtained MS/MS data were transformed from Agilent Mass Hunter data files (.d) to mzXML format via MS Convert software, a component of the ProteoWizard suite.

### Cytotoxic activity on lung cancer cell line A-549

#### Cell culture

The non-small-cell lung cancer (NSCLC) cell line (A549) and human skin fibroblast (HSF) normal cells were sourced from Nawah Scientific, Mokattam, Cairo, Egypt. Cells were cultured in Dulbecco’s Modified Eagle Medium (DMEM, Gibco-Thermo Fisher Scientific, Waltham, MA, USA; Cat. No. 11995065), complemented with 10% fetal bovine serum (FBS, Gibco-Thermo Fisher Scientific, Waltham, MA, USA; Cat. No. A5670901), and 5,000 U/mL Penicillin–Streptomycin (Gibco-Thermo Fisher Scientific, Waltham, MA, USA; Cat. No. 15070063) at 5% CO_2_ and 37 ^**◦**^C [23].

#### Evaluation of synergism

The combined effects were studied using CompuSyn software. The combination index (CI) was calculated using the median effect principle of the CompuSyn program. The software is based on the multiple drug effect equations of Chou and Talalay [24]. According to the Chou–Talalay method, CI values < 1 indicate synergism, CI = 1 indicates an additive effect, and CI > 1 indicates antagonism.

#### Cell viability assay

Cell viability was determined using the Sulforhodamine-B (SRB) assay. A549 and HSF cells were seeded in 96‐well plates, approximately 10^4^ cells/well. A549 cells were exposed to *A. vulgaris* L. (ART) aqueous extract and the standard chemotherapy cisplatin (CIS) (Sigma-Aldrich, Cat. No. 232120) concentration range (1, 10, 30, 100, and 300 µg/mL and 1, 3, 10, 30, and 100 µM, respectively). Equitoxic concentrations of ART and CIS were used in combination. Post-treatment for 72 h, cells were fixed with 150 µL of 10% trichloroacetic acid (TCA; Merck, Cat. No. 76–03–9) at 4 °C for 1 h. After washing five times with distilled water, 70 µL of 0.4% (w/v) SRB solution (Sigma-Aldrich, Cat. No. 230162) was added, and plates were incubated for 10 min at room temperature in the dark. Excess dye was removed by washing three times with 1% acetic acid (Chem-Lab, Cat. No. CL00.0106), and plates were allowed to air dry. Subsequently, 150 µL of 10 mM Tris-buffer (pH 10.5; Chem-Lab, Cat. No. CL03.0204) was added to solubilize the bound dye. Absorbance was measured at 540 nm using a BMG LABTECH® FLUOstar Omega microplate reader (Ortenberg, Germany) [25].

#### Cell cycle analysis

A549 cells were treated with the pre-calculated IC_50_ of *A. vulgaris* L. (ART) aqueous extract and cisplatin (CIS) alone or combined for 48 h. Then, cells were harvested by trypsinization, twice washed with PBS (phosphate-buffered saline) (Elabscience, Cat. No. PB180327), fixed in ice-cold 60% ethanol at 4 °C, and rewashed in PBS. After that, cells were resuspended in 500 μL propidium iodide (PI) (Sigma-Aldrich, Cat. No. P4170) with RNase (Sigma-Aldrich, Cat. No. R6148) staining buffer, BD (Franklin Lakes, NJ, USA), and incubated for 30 min. Lastly, FACS analyses were executed utilizing the ACEA Novocyte™ flow cytometer (ACEA Biosciences Inc., San Diego, USA). For every sample, data from 12,000 cells were collected, and the distribution of cell cycle phases was analyzed using ACEA Novo Express™ software (ACEA Biosciences Inc., San Diego, USA) [26].

#### Apoptosis analysis

A549 cells were treated with the pre-calculated IC_50_ of *A. vulgaris* L. (ART) aqueous extract and cisplatin (CIS) alone or combined for 48 h and then trypsinized and washed twice with PBS. Apoptosis assessment was performed via the Annexin V-FITC/PI Apoptosis Detection Kit (BD Biosciences, San Diego, USA; Cat. No. 556547), according to the manufacturer's instructions. Briefly, cells were resuspended in 0.5 mL of the binding buffer, followed by the staining solution (5 μL of Annexin V-FITC and 5 μL of PI) added for 15 min at room temperature in the dark. Finally, the cells were applied to FACS analysis using an ACEA Novocyte™ flow cytometer (ACEA Biosciences Inc., San Diego, CA, USA) [27].

#### Quantification of apoptosis-related markers using ELISA

Cells were seeded in 6-well plates and treated with A. vulgaris aqueous extract (ART), cisplatin (CIS), or their combination at their respective IC₅₀ concentrations for 48 h. Following treatment, the levels of apoptosis-related markers, including caspase-3, Bax, and Bcl-2, were quantified using ELISA according to the manufacturer's instructions. The commercial kits utilized were Invitrogen Human Caspase-3 ELISA Kit (Cat. No. KHO1091), Human Bax ELISA Kit (ab199080), and Human Bcl-2 ELISA Kit (ab119506).

#### Autophagy assessment

For autophagic assessment in response to *A. vulgaris* L. (ART) aqueous extract and cisplatin (CIS) alone or combined for 48 h, A549 cells were trypsinized and washed twice with ice-cold PBS. Then 0.5 ml of 1 μg/mL acridine orange solution (Sigma-Aldrich, Cat. No. A6014) prepared in PBS was added and incubated in the dark for 30 min at room temperature. Cells were adjusted to 12,000 events when applied to flow cytometric analysis via ACEA Novocyte™ flow cytometer, and fluorescent signals were analyzed via FL1 signal detector (488 nm excitation/530 nm emission). The net fluorescent intensities (NFI) were quantified [28].

#### Data analysis

Dose–response curves were analyzed applying the E_max_ model, and IC_50_ (half maximal inhibitory concentration) were calculated as previously described [29].Data were displayed as mean ± SD. The results were evaluated using one-way ANOVA and the Tukey–Kramer post-hoc test. All statistical analyses were performed with GraphPad Prism™ software version 9.3.1 (GraphPad Software, Inc., La Jolla, CA, USA). Statistical significance was determined at *P* ≤ 0.05.

### Molecular docking

The docking simulation was performed on 13 identified constituents on 2 apoptosis proteins **(**Bcl-2 and p53**)** and 2 autophagy proteins and autophagy (LC3C and mTOR). The crystal structures of proteins (PDB IDs: 8HOI, 3zme, 3WAM, and 9F44, respectively) were downloaded from the Protein Data Bank (PDB). Docking was performed on PyRx 0.8 software using the AutoDock Vina plugin. Water molecules, co-crystallized solvents, ligands, and other heteroatoms were removed. The internal ligand of the enzyme was redocked to validate the method. Analysis and visualization of 2D and 3D images were done using BIOVIA Discovery Studio Visualizer (v25.1.0.24284). Grid box coordinates of the binding center are collected in Table 1. To validate the docking simulation, redocking of the co-crystallized ligands was performed by the DockRMSD server [30].

**Table 1:** Grid box coordinates of binding center

Protein	Center	Size
BCL2	center_x = 18.1898125331 center_y = −2.47398042673 center_z = 40.2844085086	size_x = 10.8232250662 size_y = 15.751142782 size_z = 19.5848602585
P53	center x = 91.8542164398 center_y = 93.760458842 center_z = −45.4378819724	size_x = 15.1488826298 size_y = 19.633754203 size_z = 16.9699713015
LC3C	center_x = −39.9394450329 center_y = 10.059909222 center_z = 0.629655516001	size_x = 10.2075747754 size_y = 10.0548872889 size_z = 10.6067292767
mTOR	center_x = 308.638388266 center_y = 371.133229032 center_z = 304.837568001	size_x = 12.1001670075 size_y = 9.43419171194 size_z = 12.6134850338

## Results

### Quantitative analysis of *A. vulgaris* extract by HPLC

**Fig. 1:**
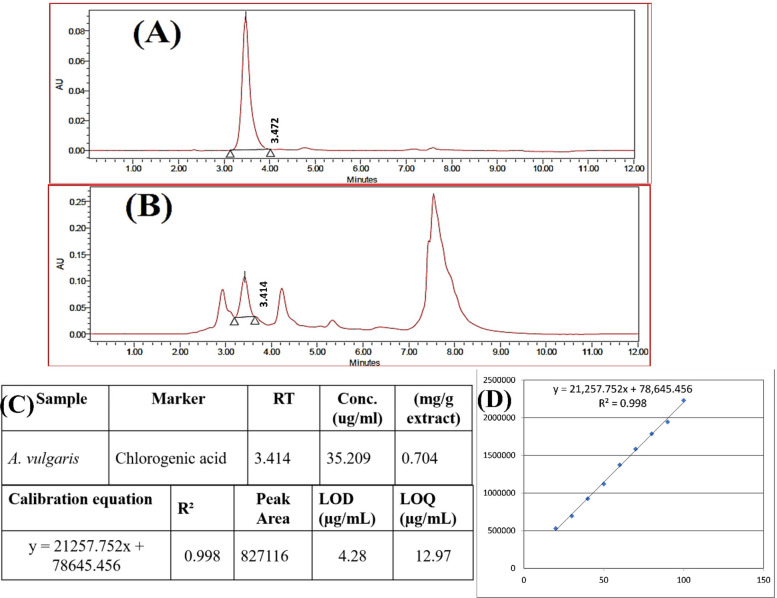
HPLC analysis of chlorogenic acid standard and *A.vulgaris* L. aqueous extract (**A**) HPLC chromatogram of chlorogenic acid standard, (**B**) HPLC chromatogram of *A. vulgaris* L. aqueous extract, (**C**) HPLC quantification results and method validation parameters for chlorogenic acid, and (**D**) calibration curve of chlorogenic acid. The calibration curve was constructed using serial dilutions of the standard, and peak areas were plotted against corresponding concentrations for quantitative analysis

A linear calibration curve of chlorogenic acid standard was prepared at a concentration range from 20 to 100 μg/mL, and the percentage of standard in the extract was determined. The content of chlorogenic acid (peak area = 827,116) in *A. vulgaris* L. was found to be 0.7 mg/g of the aqueous extract (Fig. 1). The assay was found to be sufficiently sensitive with LOD and LOQ values of 4.28 µg/mL and 12.97 µg/mL, respectively.

### Quantitative determination of total phenolic and flavonoid contents in *A. vulgaris* extract

The total flavonoid content (TFC) of the aqueous extract of *A. vulgaris* was determined using the aluminum chloride colorimetric assay and expressed as quercetin equivalents (QE). The total phenolic content (TPC) was determined using the Folin–Ciocalteu method and expressed as gallic acid equivalents (GAE). The calibration curve equations and the corresponding TFC and TPC values are presented in Table 2.

**Table 2:** Calibration curve equations and total phenolic and flavonoid contents of the aqueous extract of *A. vulgaris*

	Calibration Curve	R^2^	Content
Total flavonoid content	C.C of quercetin y = 0.003572x + 0.0837	0.9992	10.36 ± 0.81μg QE/mg extract
Total phenolic content	C.C of gallic acid y = 0.004644x + 0.01193	0.9991	93.42 ± 3.95μg GAE/mg dried extract

### UHPLC-QTOF-MS/MS qualitative profiling of *A. vulgaris* extract

**Fig. 2:**
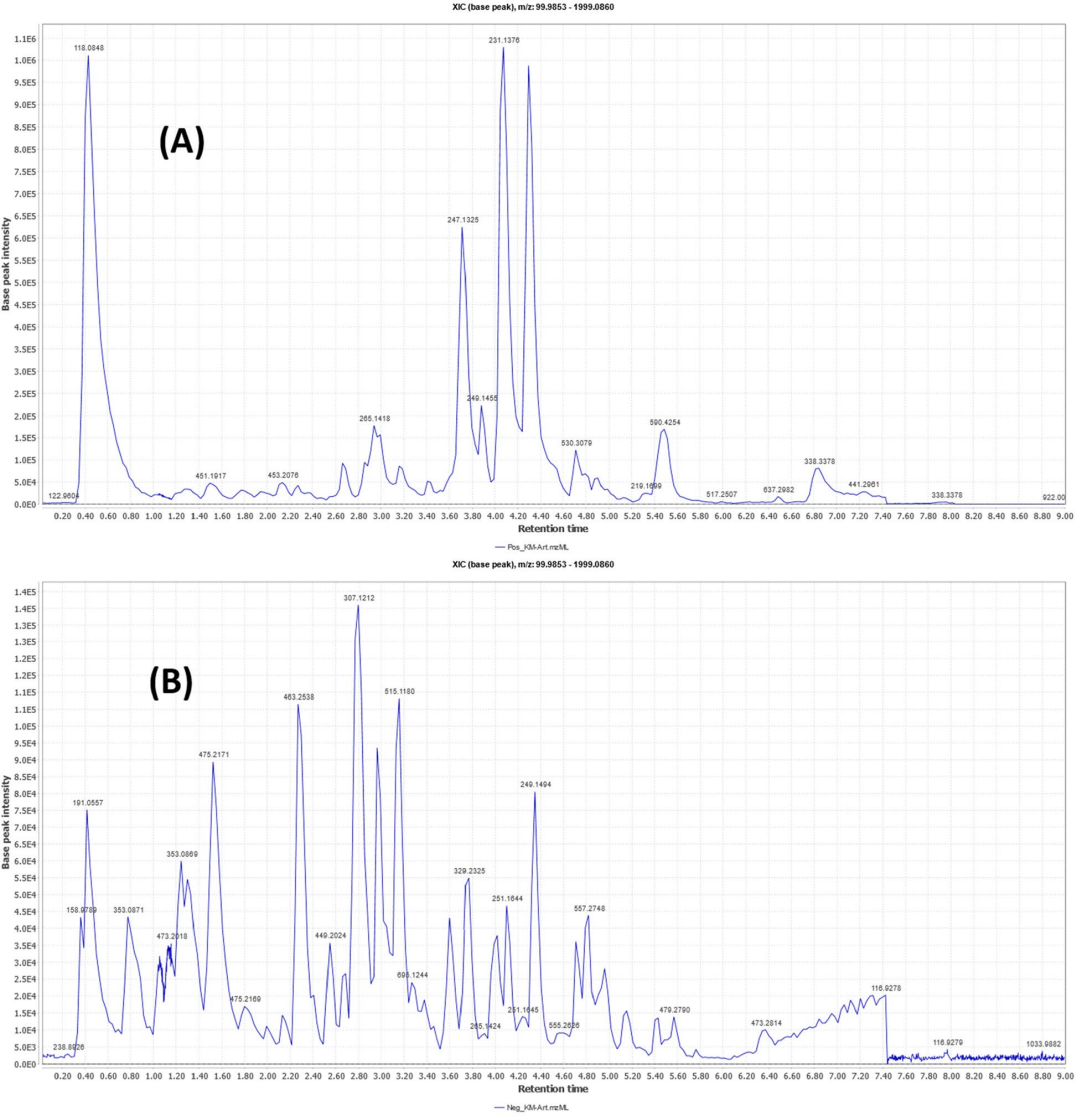
Total ion chromatograms of *A. vulgaris* L. aqueous extract in positive and negative modes (**A**) Total ion chromatograms (TIC) of *A. vulgaris* L. aqueous extract obtained using UHPLC-QTOF-MS/MS in positive and (**B**) negative ionization modes

*A. vulgaris* L. aqueous extract was analyzed in both positive and negative ion electrospray ionization (ESI) MS modes. Total ion chromatograms in positive and negative modes are presented in Fig. 2. According to the required retention time windows, accurate masses, MS/MS fragmentation patterns, and comparison with previously published data, thirty-two metabolites were tentatively identified. These comprised ten phenolic acids, three coumarins, six flavonoids, thirteen sesquiterpenes, and one fatty acid. Their mass spectrometry data are presented in Table S1 in the supplementary file. MS/MS chromatograms of all compounds mentioned at Table S1 are represented in the supplementary file, explaining their molecular fragmentation patterns (Fig. S3-S35).

#### The modulatory effect of *A. vulgaris* on cisplatin cytotoxicity in A549 cells

**Fig. 3:**
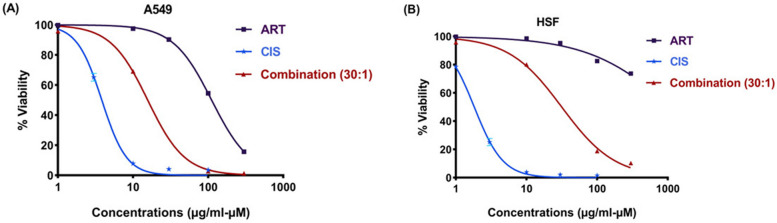
Cytotoxic and chemo modulatory effects of ART and CIS alone and in combination on A549 and HSF cells. Cytotoxic and chemo-modulatory effects of (ART) and (CIS) alone and in equitoxic combination (30:1) on (**A**) A549 lung cancer cells and (**B**) HSF normal cells following treatment for 72 h. Cell viability was evaluated by SRB assay, and data are expressed as mean ± SD (*n* = 3)

The A549 cell line was treated with *A. vulgaris* L. aqueous extract (ART) and the standard chemotherapeutic agent cisplatin (CIS) for 72 h at concentrations ranging from 1–300 μg/mL and 1–100 μM**,** respectively. The cell viability was assessed by SRB assay (Fig. 3A). Both ART and CIS showed dose-dependent cytotoxicity, with IC_50_ values of 111.41 ± 2.37 μg/ml and 3.84 ± 0.75 µM, indicating a potency ratio of around 30:1. When coupled at equitoxic ratios, ART dramatically increased the cytotoxic action of CIS, reducing the CIS IC_50_ value to 0.44 ± 0.02 μM and the ART IC_50_ value to 13.32 ± 1.66 μg/ml.

In normal HSF cells, ART exhibited low cytotoxicity, maintaining approximately 73% cell viability even at the highest tested concentration (300 μg/mL). In contrast, cisplatin (CIS) showed greater cytotoxicity on HSF cells than on A549 cells, with an IC_50_ of 1.823 μM. Notably, the combination treatment demonstrated a more favorable safety profile in HSF cells than in A549 cells, with a cisplatin IC_50_ of approximately 1 μM in HSF cells compared with 0.44 μM in A549 cells, suggesting preferential cytotoxicity toward cancer cells (Fig. 3B).

#### Evaluation of synergism

**Fig. 4:**
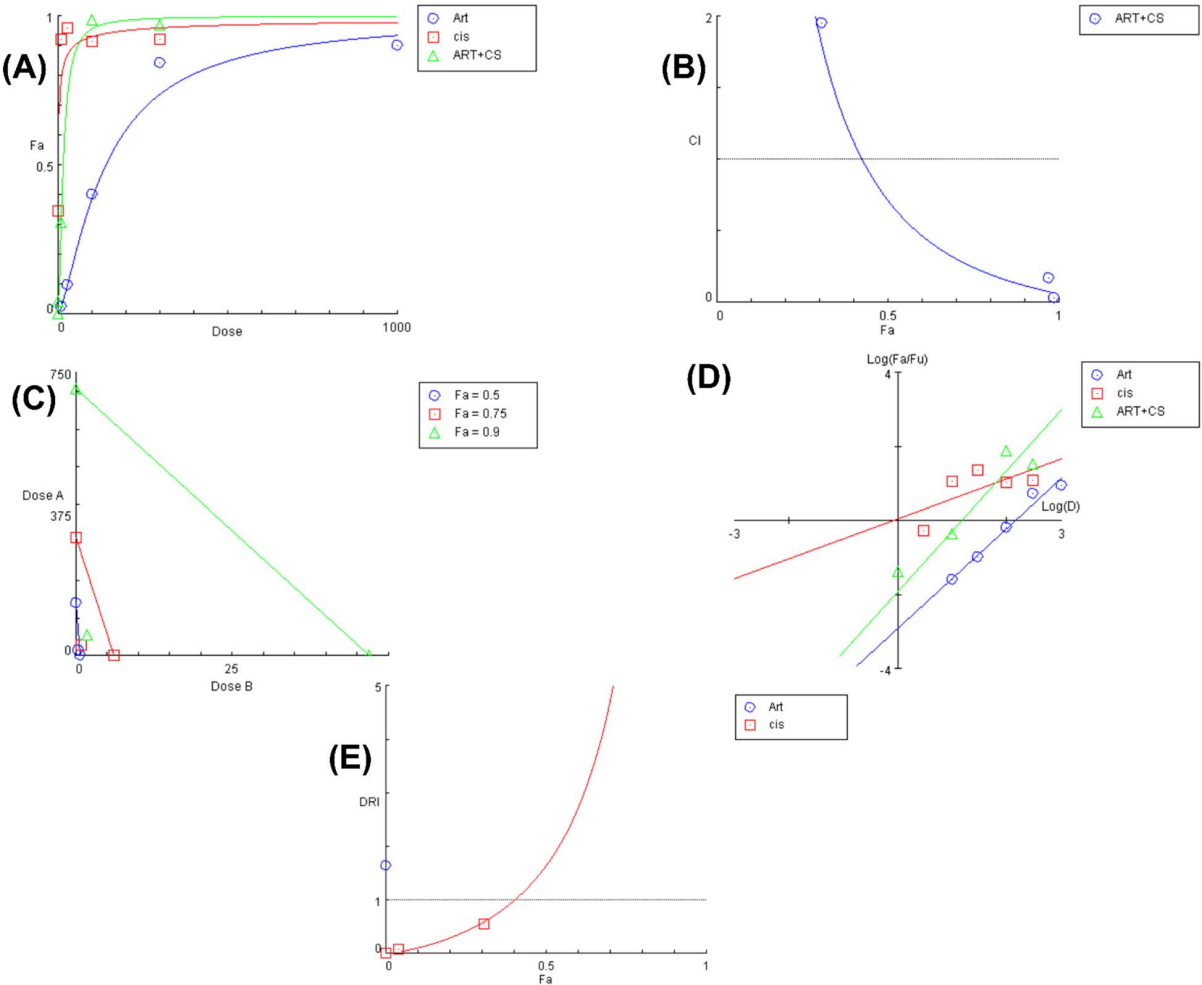
Chou–Talalay analysis of the *A. vulgaris*–cisplatin combination generated using CompuSyn software. Chou–Talalay analysis of the *A. vulgaris*–cisplatin combination generated using CompuSyn software. **A** Dose–effect curves of *A. vulgaris* extract (ART), cisplatin (CIS), and their combination at a fixed ratio of ART:CIS = 30:1. **B** Combination index (CI) plot illustrating the interaction between ART and CIS at different fraction affected (Fa) levels. **C** Isobologram analysis illustrating the interaction between ART and CIS at the fixed combination ratio of 30:1. **D** Median-effect plot generated according to the Chou–Talalay method for ART, CIS, and their combination. **E** Dose reduction index (DRI) plot showing the reduction in ART and CIS doses required to achieve equivalent effects when administered in combination

To further examine the interaction between *A. vulgaris* (ART) and cisplatin (CIS), the Chou–Talalay method was performed using CompuSyn software. The dose–effect curves, median-effect plot, combination index (CI) plot, isobologram, and dose reduction index (DRI) plot are illustrated in Fig. 4. The combination demonstrated synergistic interactions at all evaluated fraction affected (Fa) levels, with CI values below 1.0 (Table 3). At the 50% effect level (Fa = 0.50, corresponding to the IC₅₀ level), the combination showed synergism (CI = 0.711), with dose reduction indices (DRI) of 9.71 and 1.64 for ART and CIS, respectively, corresponding to dose reductions of 89.7% and 39.0%, respectively. The synergistic effect became stronger at higher Fa levels, as indicated by the progressive decrease in CI values to 0.244 at Fa = 0.75 and 0.074 at Fa = 0.97. Moreover, the DRI values (Table 3) indicate that lower doses of ART and CIS were required to achieve equivalent effects when administered in combination compared with their individual treatments. The complete CompuSyn software report is provided in Supplementary Fig. S37.

**Table 3:** Chou–Talalay analysis showing combination index (CI), predicted doses of *A. vulgaris* and cisplatin alone and in combination, and dose reduction index (DRI) values at selected fraction affected (Fa) levels

Fa	CI value	Dose of ART alone	Dose of ART in combination	Dose of CIS alone	Dose of CIS in combination	DRI (ART)	DRI (CIS)
0.5	0.711	140.07	14.43	0.79	0.48	9.71	1.64
0.75	0.244	314.72	28.25	6.09	0.94	11.14	6.46
0.97	0.074	1814.6	120.96	505.90	4.03	15.00	125.46

#### Effect of *A. vulgaris*, cisplatin, and their combination on the cell cycle distribution in A549 cells

**Fig. 5:**
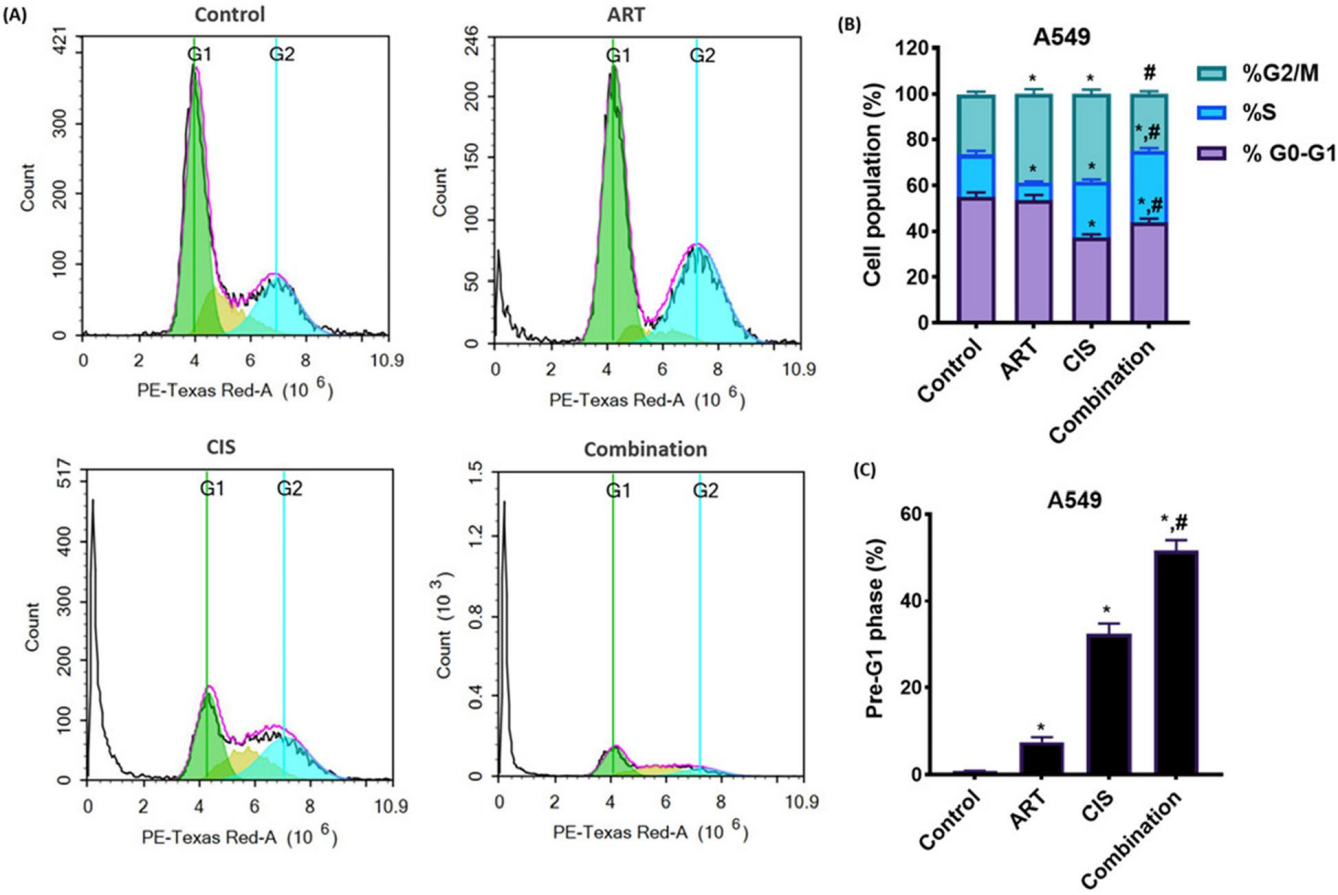
Effects of ART, CIS, and combination on A549 cell cycle distribution. **A** Cell cycle distribution in A549 cells after *A. vulgaris* L. aqueous extract (ART), cisplatin (CIS), and their combination treatment for 48 h was determined using DNA cytometry analysis compared with control (untreated cells). **B** The percentage of cells in each phase of the cell cycle was depicted as a bar graph of mean ± SD; *n* = 3. **C** The pre-G1 phase was plotted as the percentage of the total cell population. * Statistically significant from control, ^**#**^Significantly different from cisplatin group at *p* ≤ 0.05

DNA flow cytometry was used to assess the cytotoxic effect of *A.vulgaris* L. aqueous extract (ART), cisplatin (CIS) and their combination on the cell cycle distribution of the A549 lung cancer cell line (Fig. 5).

Treatment with ART showed no significant change in the cell population of the G0/G1-phase (non-proliferating cell fraction), which remained at 53.72 ± 2.18%, compared with 55.14 ± 1.9% of control cells. However, ART markedly reduced the proportion of A549 cells in the S phase (DNA synthesis phase) from 18.35 ± 1.55% in control to 7.78 ± 0.33%. Additionally, ART induced a significant G2/M phase arrest (mitotic phase), increasing the cell population in this phase from 26.5 ± 1.03% to 38.5 ± 2.18%, resulting in enhanced cell death, as manifested by elevation in the pre-G1 phase population (fragmented nuclei with diminished DNA content) from 0.82 ± 0.09% in control cells to 7.56 ± 1.11%. (CIS) treatment significantly reduced the proliferative fraction in the G0/G1 phase to 37.27 ± 1.38%. Conversely, CIS induced a notable accumulation of cells in the S-phase to 24.35 ± 1.11%, indicating cell cycle arrest and disruption of DNA synthesis in lung cancer cells (Fig. 5A, B), accompanied by a significant rise in the G2/M phase population to 38.38 ± 1.92%, suggesting mitotic arrest. These alterations in cell cycle distribution were associated with a robust increase in cell death, as reflected by a pronounced elevation in the pre-G1 phase population to 32.55 ± 2.27% (Fig. 5C).

The combination of CIS with ART exerted a very potent anti-proliferative effect compared to CIS treatment, as manifested by an increase in the cell population in the G0/G1 phase cell population from 37.27 ± 1.38% to 43.84 ± 1.72%, accompanied by cell cycle arrest in the S phase, which increased from 24.35 ± 1.11% to 31.21 ± 1.23% (Fig. 5A, B). This was further reflected in a more pronounced induction of cell death, indicated by an elevation in the pre-G1 phase from 32.55 ± 2.27% to 51.53 ± 2.44% (Fig. 5C). Reciprocally, the CIS/ART combination caused a significant decrease in the G2/M phase population, decreasing from 38.38 ± 1.92% to 24.95 ± 1.21% compared to CIS treatment.

#### Modes of cell death involved in the synergistic effect of combined treatment

To discern whether ART and CIS exert their cytotoxic and synergistic effects through apoptosis or necrosis, A549 cells were treated with the IC_50_ concentrations of ART and CIS and their combination for 48 h, and then cell death modes were assessed using PI and Annexin-V/FITC staining coupled with flow cytometry.

**Fig. 6:**
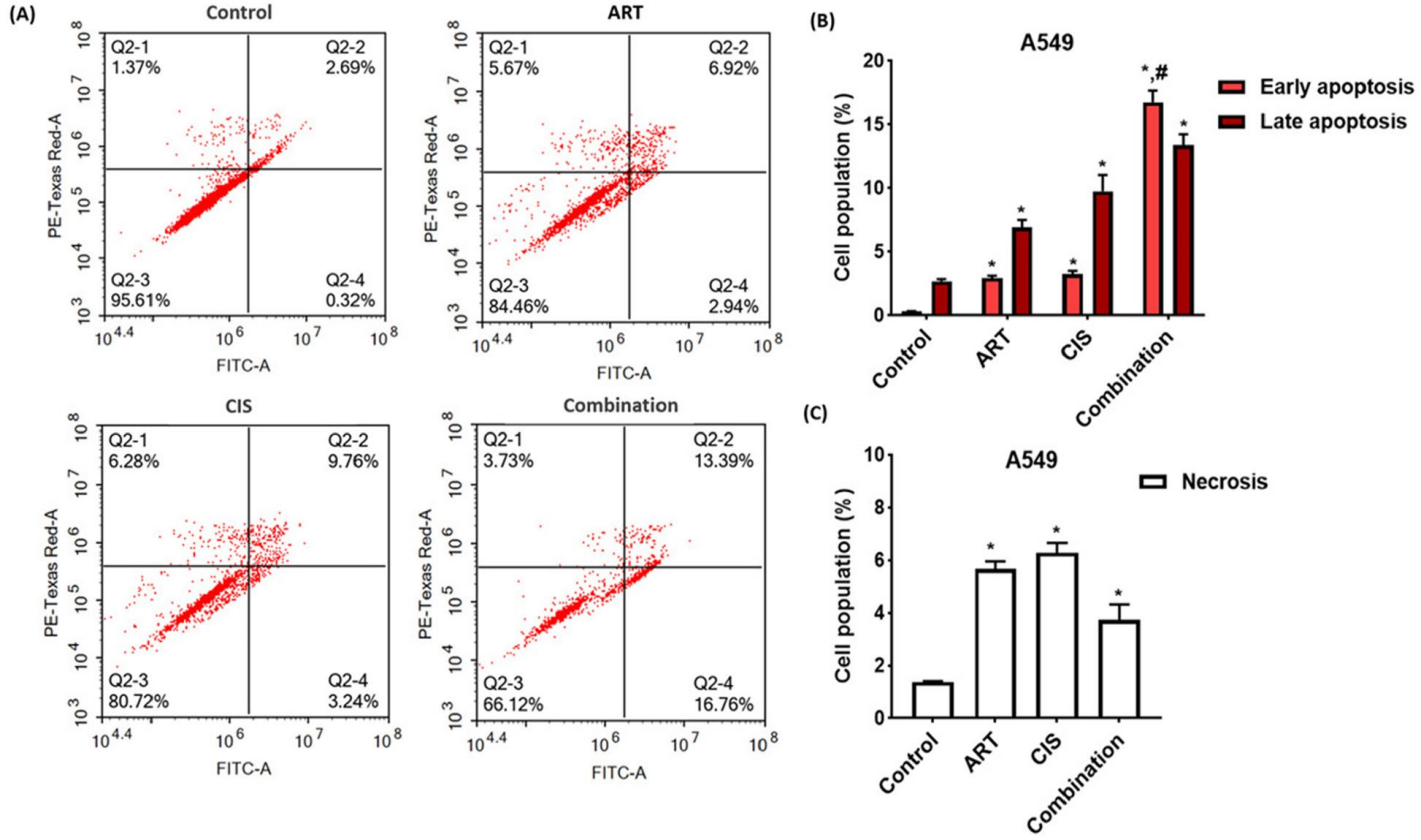
Apoptosis and necrosis in A549 cells treated with ART, CIS, and their combination. **A** Apoptosis/necrosis assessment by flow cytometry in A549 cells pretreated with IC_50_ values of *Artemisia vulgaris* L. (ART), cisplatin (CIS), and their combination for 48 h and stained with PI/Annexin V-FITC. **B** Different cell populations at early and late apoptosis and (**C**) necrosis were quantified and expressed as a percentage of total events. Data are presented as mean ± SD; *n* = 3. * Statistically significant from control, ^**#**^Significantly different from the cisplatin group at *p* ≤ 0.05

The results are outlined in Fig. 6, showing that ART and CIS triggered both modes of cell death in A549 cells, mainly through apoptotic cell death (Fig. 6A). ART treatment resulted in approximately 15% cell death, comprising 9.86 ± 0.47% apoptosis and 5.67 ± 0.28% necrosis compared to control untreated A549 cells, which showed only 3.01 ± 0.72% apoptosis and 1.37 ± 0.03% necrosis. Meanwhile, treatment with CIS resulted in around 20% total cell death, with 13 ± 0.72% apoptosis and 6.28 ± 0.37% necrosis (Fig. 6B, C).

Interestingly, compared to CIS treatment, combining CIS with ART induced further cell death primarily through apoptosis, particularly early apoptosis. The combination resulted in approximately 34% total cell death, compromising 16.76 ± 0.92% early apoptosis, 13.39 ± 0.85% late apoptosis, and only 3.73 ± 0.59% necrosis. In comparison, CIS alone induced 3.24 ± 0.26% early apoptosis, 9.76 ± 1.28% late apoptosis, and 6.28 ± 0.37% necrosis, highlighting the greater pro-apoptotic efficiency of the ART and CIS combination in A549 cells.

#### ELISA-based assessment of apoptosis-related markers

In A549 cells, treatment with ART increased caspase-3 levels to 28.78 ± 1.13, while CIS further increased it to 35.78 ± 1.80, compared with the control (19.32 ± 1.27). Notably, the combination treatment markedly elevated caspase-3 levels to 111.56 ± 1.40, indicating enhanced apoptotic activation.

Similarly, Bax expression increased from 12.25 ± 1.27 in the control group to 20.51 ± 0.99 and 33.92 ± 1.80 following ART and CIS treatment, respectively, with the highest level observed in the combination-treated group **(**77.99 ± 2.25).

**Fig. 7:**
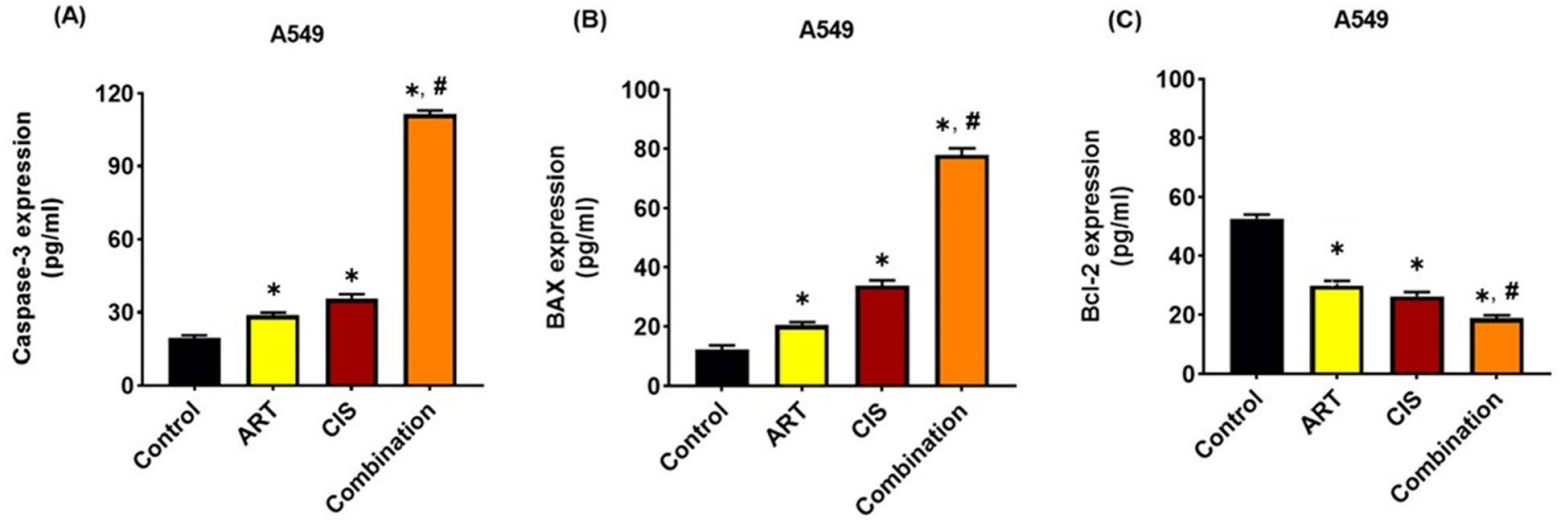
ELISA-based quantification of apoptosis-related markers in A549 cells. **A** Caspase-3, (**B**) Bax, and (**C**) Bcl-2 levels measured by ELISA in A549 cells treated with the IC₅₀ concentrations of *A. vulgaris* (ART), cisplatin (CIS), or their combination for 48 h. Data are presented as mean ± SD (*n* = 3). ^*^Statistically significant compared with the control group; ^**#**^Significantly different from the cisplatin group at *p* ≤ 0.05

In contrast, Bcl-2 levels were significantly reduced by all treatments, decreasing from 52.67 ± 1.44 in the control group to 30.02 ± 1.59 in the ART group, 26.34 ± 1.42 in the CIS group, and reaching the lowest level in the combination-treated group (18.76 ± 1.11) (Fig. 7).

#### Role of autophagy in the synergistic effect of combined treatment

**Fig. 8:**
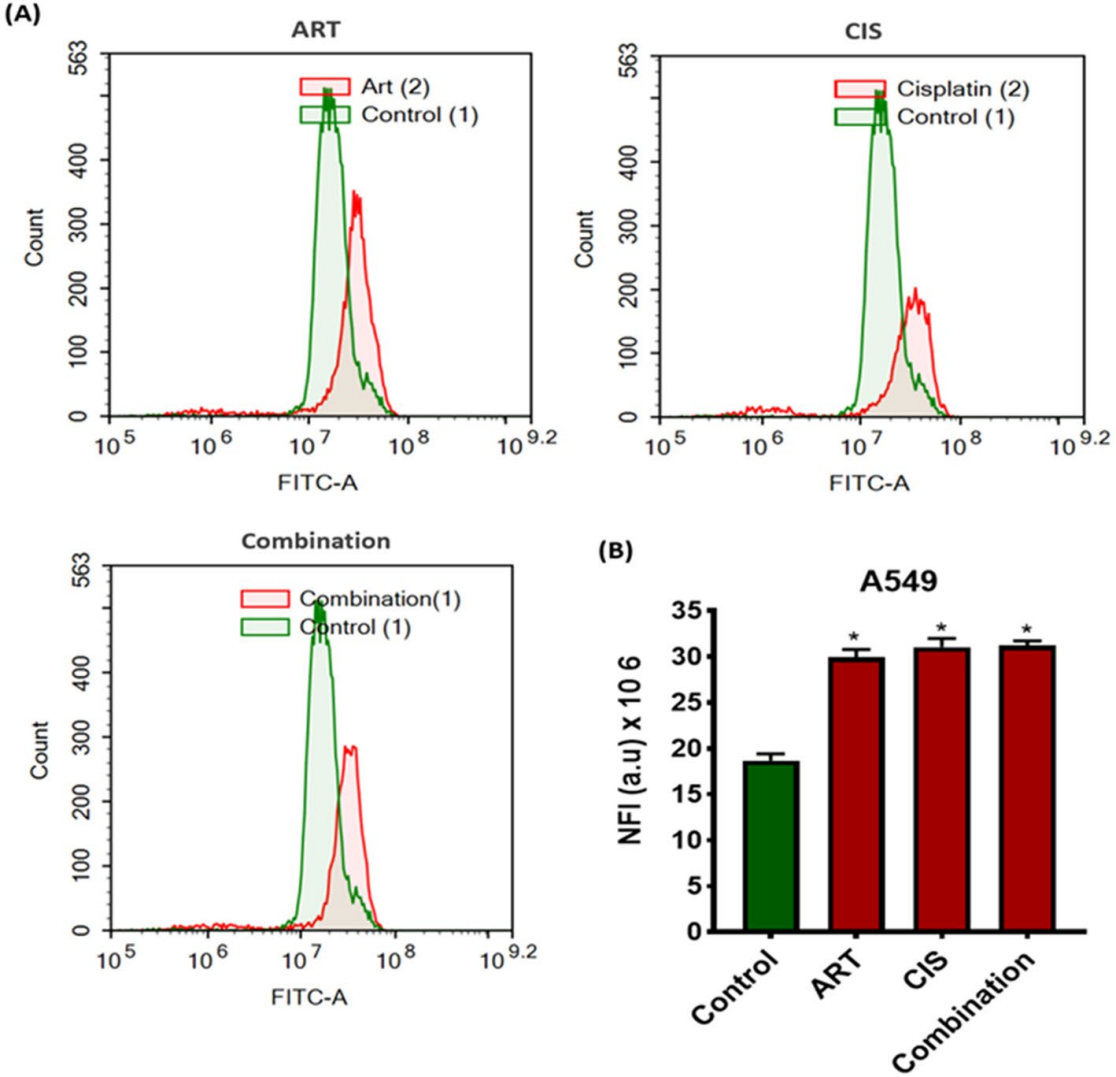
Autophagic cell death in A549 cells treated with ART, CIS, and combination. **A** Autophagic cell death evaluation in A549 cells after exposure to *A. vulgaris* (ART), cisplatin (CIS), and their combination for 48 h. Cells were stained with acridine orange dye. **B** Net fluorescent intensity (NFI) was plotted in comparison with the basal fluorescence of untreated A549 cells. Data are displayed in triplicate. * Statistically significant from control, ^**#**^Significantly different from the cisplatin group at *p* ≤ 0.05

Autophagy was evaluated in A549 lung cancer cells following 48-h treatment with ART and CIS and their combination, using acridine orange dye coupled with flow cytometry (Fig. 8).

Both ART and CIS treatments significantly increased autophagic cell death, as indicated by an increase in net fluorescent intensity (NFI) from 18.67 ± 0.75 × 10^6^ NFI of control cells to 29.94 ± 0.86 × 10^6^ NFI with ART and 31.04 ± 0.95 × 10^6^ NFI with CIS (Fig. 8A and B). Unfortunately, compared to CIS treatment, combining CIS with ART did not induce any change in autophagic signal, showing a similar NFI of 31.23 ± 0.49 × 10^6^. These findings suggest that autophagy did not play a pivotal role in mediating the synergistic effect of the CIS and ART combination.

### Molecular docking of *Artemisia vulgaris* L. active constituents with apoptosis and autophagy proteins

Thirteen compounds from the previously identified compounds in UHPLC-QTOF-MS/MS were chosen to be evaluated through molecular docking against four target proteins, including the apoptosis-related proteins (BCL2 and P53) and the autophagy-related proteins (LC3C and mTOR). Based on binding affinity values, the top three compounds exhibiting the strongest interactions with each protein were summarized in Table S2 in the supplementary file, and the whole thirteen compounds are presented in Tables S3-S7 at the end of the text. Detailed examination of their interactions with amino acid residues within the target proteins was conducted using BIOVIA Discovery Studio 2025. Hydrogen bonds were visualized using green dashed lines. Structural analysis of the ligand–protein complexes revealed multiple types of interactions, including conventional hydrogen bonds and hydrophobic interactions such as alkyl and π–alkyl bonds.

For the BCL2 apoptosis protein, the compounds aesculin, coumaroylquinic acid and luteolin exhibited the most favorable binding energies. Aesculin formed two conventional hydrogen interactions with ALA A:149 and ASN A:143, and four π–alkyl interactions with ARG A:146 (two bonds) and ALA A:149 (two bonds). Coumaroylquinic acid engaged in two conventional hydrogen bonds with ARG A:146 and TYR A:108, one π–alkyl interaction with ALA A:149, one π–donor hydrogen bond with PHE A:112 and two π– π T-shaped interactions with PHE A:104 and TYR A:108. Luteolin formed two conventional hydrogen bonds with GLY A:145 and TRP A:144, three π– π stacking interactions with TYR A:103 (two bonds) and TYR A:202, and a π–alkyl interaction with ALA A:100.

For the P53 apoptosis protein**,** aesculin**,** chlorogenic acid**,** and coumaroylquinic acid demonstrated the most favorable binding energies with hydrogen bonds. Aesculin formed eight interactions, including a conventional hydrogen bond with LEU A:145, THR A:150, THR A:230 and THR A:231, and carbon–hydrogen bond with THR A:231 and CYS A:220. Additionally, it engaged in π-alkyl interactions with CYS A:220, PRO A: 151, PRO A:223 and VAL A:147. Chlorogenic acid exhibited multiple molecular interactions within the binding site. It established four conventional hydrogen bonds with GLY A:154, LEU A:145, PRO A:152, and THR A:155. In addition, two carbon–hydrogen bonds were observed with PRO A:151 and PRO A:223. The ligand also formed two unfavorable donor–donor interactions with GLY A:154 and THR A:230, alongside a π–sigma interaction with PRO A:153 and a π–alkyl interaction with PRO A:222. Coumaroylquinic acid established conventional hydrogen bonds with ASP A:228, PRO A:219, and THR A:231. Additionally, it formed π–alkyl interactions with PRO A:223 and VAL A:147, as well as a carbon–hydrogen bond and an unfavorable donor–donor interaction with GLU A:221.

For the LC3C autophagy protein, the highest binding affinities were observed for chlorogenic acid, coumaroylquinic acid, and umbelliferone. Chlorogenic acid formed four conventional hydrogen bonds with LEU A:49, LYS A:45, LYS A:47, and SER A:65, a carbon–hydrogen bond with PRO A:51 and a π–sigma bond with ILE A:62.

Coumaroylquinic acid exhibited conventional hydrogen bonds with LEU A:49 and LYS A:45, a π–sigma bond with ILE A:62 and a π–alkyl bond with PRO A:51. Umbelliferone established two conventional hydrogen bonds with LEU A:49 and LYS A:45. In addition, it formed one π–alkyl interaction and one π–sigma interaction with ILE A:62, as well as a π–π stacking interaction with PHE A:48.

Regarding the mTOR autophagy protein, aesculin, artemisinin and rutin demonstrated the strongest binding affinities. Aesculin formed eight conventional hydrogen bonds with LYS A:1702 (two bonds), LYS A:1706, LYS A:1753, SER A:1658 (two bonds), TYR A:1698, and TRP A:1786. Additionally, it formed two π–cation interactions with LYS A:1788, and two unfavorable donor–donor interactions with LYS A:1662 and LYS A:1706. Artemisinin engaged in three conventional hydrogen bonds with ARG A:1749, LYS A:1788, and TYR A:1698, as well as a carbon hydrogen bond with ARG A:1749. Rutin established seven conventional hydrogen bonds with ARG A:1749, LYS A:1662, LYS A:1702, LYS A:1788, SER A:1658 (two bonds), and TYR A:1698. It also formed a carbon hydrogen bond and an unfavorable donor–donor interaction with ARG A:1749, in addition to a π–cation interaction with LYS A:1662. Redocking of the co-crystallized ligands was performed for docking validation. The docked conformations had RMSD values ranging from 0.724 to 2.690 Å as measured by DockRMSD server, confirming our methodology as illustrated in Table S8 at the end of the manuscript.

## Discussion

Lung cancer remains one of the most aggressive cancers and the leading causes of cancer-related mortality in both men and women [31]. Non-Small Cell Lung Cancer (NSCLC) accounts for approximately 80% of lung cancer cases, with a 5-year survival rate of only 21% [32].

Cisplatin has been a standard platinum-based anticancer drug for decades, treating patients with a variety of malignancies, including lung cancer [33]. It causes tumor death by creating covalent DNA-platinum adducts, which trigger a DNA damage response in tumour cells and activate proapoptotic pathways. Although cisplatin is highly effective in the treatment of solid tumors, drug resistance and substantial adverse effects have been reported [34]. Cisplatin combined with a chemosensitizer, or synergist is presently considered the most effective treatment for advanced NSCLC [35].

The genus *Artemisia* is a valuable source of antitumor compounds, including monoterpenoids, sesquiterpene lactones, phenolics, alkaloids, flavonoids, terpenoids, steroids, and essential oils [36, 37].

HPLC investigation revealed the presence of chlorogenic acid, a derivative of caffeoylquinic acid, in the aqueous extract of *A. vulgaris* and estimated its amount as 0.7 mg/g of extract. This finding is consistent with previous research that found chlorogenic acid to be one of the primary phenolic compounds in *A. vulgaris* and other species of the genus *Artemisia*. Chlorogenic acid has been reported to have antioxidant and anticancer activities and may contribute to some of the biological activity of the extract. Additionally, its identification and quantification are valuable chemical markers for quality assessment and standardization of the aqueous extract. The low LOD and LOQ values also indicate the sensitivity of the developed HPLC method.

Total phenolic and total flavonoid assays revealed a higher total phenolic content (93.42 ± 3.95 μg GAE/mg dry extract) than total flavonoid content (10.36 ± 0.81 μg QE/mg extract) in the aqueous extract of *A. vulgaris*. This suggests that flavonoids only constitute a small percentage of the polyphenolic fraction. This result is consistent with the LC–MS/MS analysis in this study, which demonstrated a larger number of phenolic compounds other than flavonoids; such as phenolic acids and coumarins. These compounds could be responsible for the antioxidant and anticancer properties observed in the present study.

UHPLC-QTOF-MS/MS analysis of phytochemicals in *Artemisia vulgari****s*** L. aqueous extract revealed a wide array of phenolic acid derivatives, particularly quinic acid [38–40] derivatives, which were tentatively identified in *A. vulgaris* L. based on LC–MS/MS fragmentation patterns and supported by literature comparisons. Coumaroylquinic acid [38, 41, 42] was detected in negative ionization mode at *m/z* 337.0923 [M − H]⁻, with a fragment at *m/z* 191.0552, indicative of the loss of the coumaroyl group and confirming the presence of quinic acid, which is characterized by a base peak at *m/z* 191 [quinic acid − H]⁻. Additional fragments at *m/z* 163.0398 and *m/z* 119.0500 suggested sequential losses of formyl and carboxylic acid groups, respectively. Caffeoylquinic acid [38–40, 43–45] appeared at *m/z* 353.0867 [M − H]⁻ with key product ions at *m/z* 191.0555, consistent with caffeoyl group loss, and *m/z* 179.0377, which corresponds to deprotonated caffeic acid ([caffeic acid − H]⁻) [44]. Further fragmentation yielded *m/z* 135.0445 (loss of the carboxyl group) and *m/z* 173.0445, 155.0346, and 137.0231, attributed to consecutive losses of water. Feruloylquinic acid [39, 44] was identified at m/z 367.1025 [M − H]⁻, and its MS/MS spectrum showed fragment ions at *m/z* 191.0556 (quinic acid moiety) and *m/z* 173.0447 (loss of water), as well as *m/z* 129.0185 (loss of the carboxylic acid group). Dicaffeoylquinic acid [38, 39, 42, 44] (isochlorogenic acid) was detected at *m/z* 515.1184 [M − H]⁻, producing major fragments at *m/z* 353.0869 (loss of one caffeoyl moiety) and *m/z* 191.0554 (further loss of the second caffeoyl group, revealing quinic acid). Additional fragments at *m/z* 173.0449 and 155.0339 were attributed to dehydration, while *m/z* 111.0449 suggested the loss of a carboxylic group.

A related compound at *m/z* 499.1239 [M − H]⁻ was identified with fragments at *m/z* 191.0554 (quinic acid), *m/z* 179.0340 (caffeic acid) [40, 44, 45], *m/z* 173.0436 (loss of quinic acid and water), and *m/z* 163.0399 (coumaric acid), suggesting the presence of a coumaroylcaffeoylquinic acid isomer [42, 46]. Another high molecular weight compound was detected at *m/z* 695.1241 [M − H]⁻, with fragments at *m/z* 533.2166, 371.0580, and 209.0297, consistent with the sequential loss of three caffeoyl units ([M − H − 3 × 162]⁻), and m/z 191.0186 confirming the quinic acid core. The final fragment at *m/z* 147.0289 reflects the loss of a carboxylic acid group, supporting its tentative identification as a tricaffeoylhexaric acid isomer [46].

Three coumarin compounds were tentatively identified in the *A. vulgaris* L. aqueous extract using UHPLC-QTOF-MS/MS in negative and positive ion modes, based on their molecular ions and characteristic fragmentation patterns. These included aesculin [40, 42], umbelliferone [42, 47] and aesculetin [42, 48].

In positive ion mode, umbelliferone was detected at *m/z* 163.0387 [M + H]⁺, with a daughter ion at *m/z* 135.0418, confirming formyl loss, and another ion at *m/z* 117.0306, suggesting the loss of a water molecule. Further fragmentation yielded *m/z* 89.0379, potentially indicating loss of both water and formyl groups ([M + H − H₂O − CO]⁺). Aesculetin, another well-documented coumarin in the *Artemisia* genus [42], was detected at *m/z* 177.0187 [M − H]⁻. Its fragmentation included a peak at *m/z* 149.0214 and another at *m/z* 105.0340 likely corresponding to the loss of two formyl groups. Aesculin, a known coumarin glucoside reported in *Artemisia* species [42], was detected at *m/z* 339.0712 [M − H**]⁻**. Its product ion spectrum revealed a fragment at *m/z* 177.0181, indicating the loss of an O-hexose group ([M − H − 162]⁻), while a subsequent fragment at *m/z* 133.0290 suggested the loss of a carboxylic group ([M − H − 162 − 44]⁻). These coumarins, characterized by their fragmentation through typical neutral losses such as 162 amu (O-hexose), 44 amu (carboxylic group), and 28 amu (formyl group), reflect the structural diversity of coumarins present in *A. vulgaris* L.

Flavonoid content in *A. vulgaris* L. was found to be relatively low compared to other *Artemisia* species, consistent with previous reports [46]. Nonetheless, several flavonoid derivatives were tentatively identified by LC–MS/MS in negative ionization mode. Their fragmentation pathways provided insight into their structural features, particularly the nature of glycosidic linkages and aglycone types.

C-glycosylated flavonoids exhibited characteristic neutral losses such as [M–90–H]⁻, [M–120–H]⁻, [M–60–H]**⁻**, and [M–104–H]**⁻**, consistent with sugar moiety cleavages. Fragmentation of C-glycosides generally involves the following: C-hexosides: loss of 90 and 120 amu, C-pentosides: loss of 60 and 90 amu, and C-deoxyhexosides: loss of 74 and 104 amu.

In contrast, O-glycosylated flavonoids show typical neutral losses such as 162 amu (hexose) and 146 amu (rhamnose), indicative of glycosidic bond cleavage [49].

Quercetin-3-O-glucoside (Hyperoside) [39, 41] was detected at *m/z* 463.0871 [M − H]⁻, with a prominent fragment at *m/z* 301.0337, corresponding to the quercetin aglycone following hexose (glucose) cleavage ([M–H–162]⁻). A compound was tentatively characterized as vicenin 2 [41], detected at *m/z* 593.1494 [M − H]⁻. The MS/MS fragmentation spectrum revealed product ions at *m/z* 503.1151 and *m/z* 473.1050, corresponding to the successive losses of a C-hexoside moiety ([M–90–H]⁻ and [M–120–H]⁻). Further fragmentation produced ions at *m/z* 383.0710 and *m/z* 353.0647, which similarly indicated the loss of an additional C-hexoside unit.

*Artemisia* species are known to be rich in sesquiterpene lactones, and the most recognized compound is artemisinin, which is isolated from *A. annua* [50]. In the current analysis, artemisinin [10, 51, 52] was identified in the negative ionization mode at *m/z* 281.1384 [M − H]⁻, producing a characteristic fragment at *m/z* 263.0142, which corresponds to the loss of a water molecule ([M − H − H₂O]⁻). Further fragmentation generated a peak at *m/z* 219.1386, indicative of the loss of a carboxyl group ([M − H − H₂O − COO]⁻). In the positive ionization mode, artemisinin was detected as a dehydrated ion at *m/z* 265.1432 [M + H − H₂O]⁺. The MS/MS spectrum revealed daughter ions at *m/z* 247.1270, 229.1200, and 211.1074, corresponding to successive water losses, alongside a fragment at *m/z* 201.1272, which may also indicate the elimination of a formyl group. Another sesquiterpene, costunolide [47, 53], was detected in positive mode at *m/z* 233.1532 [M + H]**⁺**, producing a product ion at *m/z* 215.1427, indicating the loss of a water molecule, *m/z* 187.1465, indicating loss of a formyl group, and *m/z* 161.1318 and 145.0969, consistent with the sequential loss of methyl groups. Among the sesquiterpenes, vulgarin, which is commonly found in *A. vulgaris* [42], was detected in positive mode. It appeared at *m/z* 265.1432 [M + H]**⁺**, with product ions at *m/z* 247.1270 and 229.1200 (loss of two water molecules), and further fragmentation leading to *m/z* 201.1272 and 173.0566, which were attributed to the loss of two formyl groups, and *m/z* 131.0843, suggesting the loss of three methyl groups. Santonin [42, 47, 54], a well-known sesquiterpene lactone in *Artemisia* species, was detected at *m/z* 247.1328 [M + H]⁺. The MS/MS spectrum showed a prominent fragment at *m/z* 229.1200, corresponding to the loss of a water molecule. Additional product ions at *m/z* 201.1223 and 173.0932 reflected the sequential loss of formyl groups ([M + H–H₂O–CO–CO]⁺). Further fragmentation produced ions at *m/z* 159.0798, 145.0969, and 131.0843, suggesting stepwise eliminations of methyl groups. In summary, the sesquiterpenes identified in *A. vulgaris* displayed characteristic MS/MS fragmentation pathways, involving the loss of methyl groups, water, formyl, and carboxyl moieties, according to the structural behavior.

SRB assay results showed that the extract and cisplatin suppressed A549 cell proliferation in a dose-dependent manner. These findings agree with previous studies that have reported its cytotoxic activity against various human cancer cell lines, highlighting *Artemisia* as a promising plant for cancer therapy [55–57].

*Artemisia* and its constituents have also been used as chemosensitizers for conventional treatments and as radiosensitizers in various drug-resistant cancer cell lines [58–60]. The IC_50_ values obtained in the present study were much lower than those of the individual treatment, showing a considerable synergistic effect of the combination of cisplatin and the extract of *A. vulgaris* on A549 lung cancer cells. This synergistic interaction was further confirmed by Chou–Talalay analysis, with CI values less than 1.0 at all Fa levels examined, suggesting true synergism rather than an additive effect. Furthermore, analysis of the DRI revealed that a lower dose of both *A. vulgaris* and cisplatin was necessary to achieve equal therapeutic efficacy when given in combination. The results indicated that A. vulgaris increased the sensitivity of lung cancer cells to cisplatin and may be a possible adjuvant approach in the treatment of NSCLC.

Anticancer drugs kill tumor malignant cells mainly by apoptosis and trigger cell cycle arrest to attenuate the tumor cell growth [61, 62]. Therefore, we investigated the effect of the ART extract on the cell cycle dynamics of non-small lung cancer cells (A549). ART treatment resulted in substantial G2/M phase arrest. This checkpoint is critical for stopping cells from starting mitosis with damaged DNA, thus providing an opportunity to repair or remove the damage [63].

Interestingly, when ART was combined with CIS, the treatment showed a pronounced antiproliferative effect, resulting mainly in a G0/G1 phase arrest. This finding is consistent with previous results, reporting the G0/G1 phase arrest as a key mechanism of its anticancer activity [60, 62]. The observed induction of cell death, demonstrated by a robust increase in the pre-G1 phase, explains to some extent the observed synergistic activity in the cytotoxicity test of the combined treatment.

One of the key mechanisms for preventing tumorigenesis is the execution of apoptosis to malignant cells [19]. Most chemotherapeutic drugs promote apoptosis in response to intercellular death signals triggered by DNA damage or oncogenic activation [64, 65]. In the present study, ART induced apoptotic cell death rather than necrosis. These results are consistent with previous findings showing that ART exhibited apoptosis-inducing activity by arresting the cell cycle at the G0/G1 phase [59]. Furthermore, ART significantly enhanced cisplatin-induced apoptotic cell death, highlighting the therapeutic potential of this combination in lung cancer management, particularly in overcoming the ability of cancer cells to evade apoptosis [66].

Apoptosis has two pathways: extrinsic and intrinsic. Activation of Caspase-8 initiates the extrinsic pathway by activating the executioner caspase-3, whereas members of the Bcl-2 family, such as the pro-apoptotic protein Bax and the anti-apoptotic protein Bcl-2, regulate the intrinsic mitochondrial pathway [67]. In the present study, the combination treatment markedly increased caspase-3 and Bax protein levels while decreasing Bcl-2 levels, indicating enhanced activation of the apoptotic pathway. These findings support the conclusion that the observed synergistic anticancer effect is mediated predominantly by apoptosis.

Most studies on *Artemisia* species have focused on their pro-apoptotic properties; therefore, the present study also evaluated the effect of ART on cellular autophagy. Previous reports have demonstrated that Artemisia extracts can induce autophagy in several cancer models, including ovarian and prostate cancer cells [58, 67]. Consistent with these observations, our results showed that ART triggered cellular autophagy in the lung cancer A549 cell line. However, ART did not enhance the sensitivity of the A549 cells to cisplatin-induced autophagic cell death. Although the combination treatment did not improve autophagy compared with cisplatin alone, it significantly potentiated apoptotic cell death, particularly early apoptosis. Therefore, the observed synergistic reduction in cell viability is most likely mediated predominantly through apoptosis rather than increased autophagic activity.

Based on previous results demonstrating the ability of *A. vulgaris* to exert cytotoxic activity, molecular docking was performed by using 13 compounds belonging to four different chemical classes against four targeted proteins involved in both apoptosis and autophagy pathways. The docking results suggested potential interactions between several identified compounds and the selected proteins, with favorable binding affinities and multiple hydrogen-bonding interactions. Phenolic acids (chlorogenic acid and coumaroylquinic acid), flavonoids (luteolin), and the coumarin (aesculin) exhibited notable interactions with apoptosis-related targets (BCL2 and P53), while coumarins (aesculetin, aesculin, and umbelliferone), the sesquiterpene artemisinin, and the flavonoid rutin showed favorable predicted binding toward autophagy-related proteins (LC3 and mTOR). These findings provide supportive evidence for the possible involvement of these compounds in the observed biological activities; however, further experimental validation is required to confirm their molecular targets and mechanisms of action.

## Conclusion

This study demonstrates that *A. vulgaris* L. aqueous extract exhibits significant cytotoxic effects on A549 lung cancer cells, both alone and in combination with cisplatin. The combination treatment was more effective than either treatment alone, suggesting that the two drugs might act synergistically to minimize the dose of cisplatin and associated side effects. The LC–MS/MS analysis revealed that *A. vulgaris* L. possesses a diverse array of phytochemicals, including phenolic compounds, sesquiterpene lactones, coumarins, and flavonoids. Many of these compounds have been reported to possess anticancer activity. The synergistic anticancer activity was predominantly mediated through apoptosis rather than enhanced autophagy. Molecular docking suggested favorable binding interactions between several identified compounds and the selected target proteins. These data suggest that *A. vulgaris* may be used as a complementary treatment for lung cancer. The present findings are limited to the A549 cell line and should be validated in other cancer models in future studies.

## Supplementary Information

Supplementary Material 1.

Supplementary Material 2.

Supplementary Material 3.

## Data Availability

All data generated or analyzed during this study are included in this published article and its supplementary information files.
