# Supplementary material for: Synergistic anti-cancer activity of Artemisia vulgaris L. aqueous extract with cisplatin against A549 lung cancer cell line
Source: BMC Complement Med Ther. 2026 Aug 11;26:242. doi: 10.1186/s12906-026-05481-5 (PMC13459383; doi:10.1186/s12906-026-05481-5)
Supplement: Supplementary file 1 [file 12906_2026_5481_MOESM1_ESM.docx]

**Synergistic anti-cancer activity of *Artemisia vulgaris L.* aqueous extract with cisplatin against A549 Lung cancer cell line**

**Seham Salah El-Din Elhawary^1^, Hadeel Nabil Ahmed^1, *^, Mouchira A. Choucry ^2,1^, Rasha M. Allam^3^, Osama G. Mohamed^1,4^, Kamel Mahmoud Kamel^5 #^, Amira K. Elmotayam^1*, #^**

^1^ Department of Pharmacognosy, Faculty of Pharmacy, Cairo University, Kasr el-Aini Street, Cairo, 11562, Egypt

^2^ Department of Pharmacognosy, Faculty of Pharmacy and Drug Technology, Egyptian Chinese University, 11786 Cairo, Egypt

^3^ Pharmacology Department, Medical Research Institute, National Research Center, 33 El-Bohouth St., Dokki, P.O.12622, Cairo, Egypt.

^4^ Natural Products Discovery Core, Life Sciences Institute, University of Michigan, Ann Arbor, MI 48109, USA

^5^ Department of pharmacognosy, Faculty of Pharmaceutical Sciences & Drug Manufacturing, Misr University for Science and Technology, Giza, Egypt

^*^Corresponding authors emails: hadeelnabil248@gmail.com, [Amira.kamal@pharma.cu.edu.eg](mailto:Amira.kamal@pharma.cu.edu.eg)

^#^ The last two authors contributed equally to this work

**Figure S1**: MS/MS chromatogram of quinic acid

 MS/MS chromatogram of quinic acid detected in A. vulgaris aqueous extract under negative ionization mode using UHPLC-QTOF-MS/MS.

**Figure S2**: MS/MS chromatogram of chlorogenic acid

MS/MS chromatogram of chlorogenic acid identified in A. vulgaris aqueous extract under negative ionization mode.

**Figure S3:** MS/MS chromatogram of dihydroxybenzoic acid hexoside isomer

MS/MS chromatogram of dihydroxybenzoic acid hexoside isomer detected in *A. vulgaris* aqueous extract under negative ionization mode.

**Figure S4:** MS/MS chromatogram of dihydroxy benzoic acid

MS/MS chromatogram of dihydroxybenzoic acid identified in A. vulgaris aqueous extract under negative ionization mode.

**Figure S5:** MS/MS chromatogram of caffeic acid

MS/MS chromatogram of caffeic acid detected in A. vulgaris aqueous extract under negative ionization mode.

**Figure S6:** MS/MS chromatogram of coumaroylquinic acid

MS/MS chromatogram of coumaroylquinic acid identified in *A. vulgaris* aqueous extract under negative ionization mode.

**Figure S7:** MS/MS chromatogram of feruloylquinic acid

MS/MS chromatogram of feruloylquinic acid detected in A. vulgaris aqueous extract under negative ionization mode.

**Figure S8:** MS/MS chromatogram of dicaffeoylquinic acid

MS/MS chromatogram of dicaffeoylquinic acid identified in A. vulgaris aqueous extract under negative ionization mode.

**Figure S9**: MS/MS chromatogram of tricaffeoylhexaric acid isomer

MS/MS chromatogram of tricaffeoylhexaric acid isomer detected in A. vulgaris aqueous extract under negative ionization mode.

**Figure S10:** MS/MS chromatogram of coumaroylcaffeoylquinic acid isomer

MS/MS chromatogram of coumaroylcaffeoylquinic acid isomer identified in A. vulgaris aqueous extract under negative ionization mode.

**Figure S11**: MS/MS chromatogram of aesculin

MS/MS chromatogram of aesculin detected in A. vulgaris aqueous extract under negative ionization mode.

**Figure S12:** MS/MS chromatogram of umbelliferone

MS/MS chromatogram of umbelliferone identified in A. vulgaris aqueous extract under positive ionization mode.

**Figure S13**: MS/MS chromatogram of aescultin

MS/MS chromatogram of aesculetin detected in *A. vulgaris* aqueous extract under negative ionization mode.

**Figure S14**: MS/MS chromatogram of Vicenin 2

MS/MS chromatogram of vicenin II identified in A. vulgaris aqueous extract under negative ionization mode.

**Figure S15:** MS/MS chromatogram of rutin

MS/MS chromatogram of rutin identified in A. vulgaris aqueous extract under negative ionization mode.

**Figure S16**: MS/MS chromatogram of hyperoside

MS/MS chromatogram of hyperoside identified in A. vulgaris aqueous extract under negative ionization mode.

**Figure S17:** MS/MS chromatogram of luteolin

MS/MS chromatogram of luteolin identified in A. vulgaris aqueous extract under negative ionization mode.

**Figure S18:** MS/MS chromatogram of isorhamntin

MS/MS chromatogram of isorhamntin identified in A. vulgaris aqueous extract under negative ionization mode.

**Figure S19:** MS/MS chromatogram of apigenin

MS/MS chromatogram of apigenin identified in A. vulgaris aqueous extract under negative ionization mode.

**Figure S20:** MS/MS chromatogram of psilostachyin

MS/MS chromatogram of psilostachyin identified in A. vulgaris aqueous extract under positive ionization mode.

**Figure S21:** MS/MS chromatogram of vulgarolide

MS/MS chromatogram of vulgarolide identified in A. vulgaris aqueous extract under positive ionization mode.

**Figure S22:** MS/MS positive chromatogram of artemisinin

MS/MS chromatogram of artemisinin identified in A. vulgaris aqueous extract under positive ionization mode.

**Figure S23:** MS/MS negative chromatogram of artemisinin MS/MS chromatogram of artemisinin identified in A. vulgaris aqueous extract under negative ionization mode.

**Figure S24**: MS/MS chromatogram of vulgarin

MS/MS chromatogram of vulgarin identified in A. vulgaris aqueous extract under positive ionization mode.

**Figure S25:** MS/MS chromatogram of artemisin

MS/MS chromatogram of artemisin identified in A. vulgaris aqueous extract under positive ionization mode.

**Figure S26:** MS/MS chromatogram of artemin

MS/MS chromatogram of artemin identified in A. vulgaris aqueous extract under positive ionization mode.

**Figure S27**: MS/MS chromatogram of artemisinic acid

MS/MS chromatogram of artemisinic acid identified in A. vulgaris aqueous extract under positive ionization mode.

**Figure S28:** MS/MS chromatogram of arteannuin b

MS/MS chromatogram of arteannuin b identified in A. vulgaris aqueous extract under positive ionization mode.

**Figure S29**: MS/MS chromatogram of α-epoxydihydroartemisinic

MS/MS chromatogram of α-epoxydihydroartemisinic identified in A. vulgaris aqueous extract under negative ionization mode.

**Figure S30**: MS/MS chromatogram of santonin

MS/MS chromatogram of santonin identified in A. vulgaris aqueous extract under positive ionization mode.

**Figure S31:** MS/MS chromatogram of costunolide

MS/MS chromatogram of costunolide identified in A. vulgaris aqueous extract under positive ionization mode.

**Figure S32:** MS/MS chromatogram of dihydroartemisinic acid

MS/MS chromatogram of dihydroartemisinic acid identified in A. vulgaris aqueous extract under negative ionization mode.

**Figure S33:** MS/MS chromatogram of linolenic acid

MS/MS chromatogram of linolenic acid identified in A. vulgaris aqueous extract under positive ionization mode.

**Table S1: Secondary metabolites identified in *Artemisia vulgaris* aqueous extract using UHPLC-QTOF-MS/MS in negative and positive mode.**

| **Peak.** | **RT** | **Molecular formula** | ***m/z* (–)**  **detected** | **Mass error (ppm)** | **MS fragments (*m*/*z*)** | **[M−H]−/ [M+H]+, m/z** | **Identification** |
| --- | --- | --- | --- | --- | --- | --- | --- |
| **Phenolic acids** | | | | | | | |
| 1 | 0.4 | C_7_H_12_O_6_ | 191.0556 | **-**2.68 | 173.0448/127.0398/93.0342/ 85.0295/59.0138 | [M−H]− | Quinic acid |
| 2 | 0.78 | C_16_H_18_O_9_ | 353.0867 | -3.13 | 191.0555/179.0345/173.0445/ 161.0226/155.0346/137.0231/135.0445/129.0551/111.0435/93.0335 | [M−H]− | Caffeoylquinic acid |
| 3 | 0.8 | C_13_H_16_O_9_ | 315.0715 | -2.08 | 153.0187/109.0283/108.0204 | [M−H]− | Dihydroxybenzoic  acid hexoside isomer |
| 4 | 0.83 | C_7_H_6_O_4_ | 153.0188 | -3.48 | 109.0289/108.0203 | [M−H]− | Dihydroxy benzoic acid |
| 5 | 1.54 | C_9_H_8_O_4_ | 179.0344 | -2.69 | 135.0445/134.0361/107.0485/79.0553 | [M−H]− | Caffeic acid |
| 6 | 2.2 | C_16_H_18_O_8_ | 337.0921 | -2.35 | 191.0552/173.0443/163.0398/119.0500 /97.0273/93.0338/85.0291 | [M−H]− | Coumaroylquinic acid |
| 7 | 2.4 | C_17_H_20_O_9_ | 367.1025 | -2.6 | 191.0556/173.0447/129.0185/93.0337 | [M−H]− | Feruloylquinic acid |
| 8 | 3.17 | C_25_H_24_O_12_ | 515.119 | -2.13 | 353.0869/191.0554/173.0449/161.0241/137.0235/135.0443 /111.0449 | [M−H]− | DiCaffeoylquinic acid |
| 9 | 3.28 | C_33_H_28_O_17_ | 695.1241 | -1.83 | 533.2166/371.0580/209.0297 | [M−H]− | Tricaffoylhexaric acid |
| 10 | 3.41 | C_25_H_24_O_11_ | 499.1239 | -1.37 | 191.0554/179.0340/173.0436/163.0399 | [M−H]− | Coumaroylcaffeoylquinic acid |
| **Coumarins** | | | | | | | |
| 11 | 1 | C_15_H_16_O_9_ | 339.0712 | -1.93 | 295.0557/177.0818/148.0147/133.0290 | [M−H]− | Aesculin |
| 12 | 1.31 | C_9_H_6_O_3_ | 163.0387 | -1.66 | 145.0258/135.0418/117.0306/107.0463/89.0379/79.0525 | [M+H]+ | Umbelliferenone |
| 13 | 1.53 | C_9_H_6_O_4_ | 177.0187 | -3.01 | 177.0184/149.0214 /133.0290/105.0340/ 89.0393 | [M−H]− | Aesculetin |
| **Flavonoids** | | | | | | | |
| 14 | 2.27 | C_27_H_30_O_15_ | 593.1494 | -3.02 | 503.1151/473.1050/397.0354/383.0710/365.0205/353.0647/325.0691 | [M−H]− | Vicenin 2 |
| 15 | 2.73 | C_27_H_30_O_16_ | 609.1445 | -2.64 | 609.1425/301.0339/300.0257 | [M−H]− | Rutin |
| 16 | 2.85 | C_21_H_20_O_12_ | 463.0877 | -2.73 | 301.0337/300.0271/271.0239/255.0287/178.9952 /151.0006 | [M−H]− | Hyperoside |
| 17 | 3.58 | C_15_H_10_O_6_ | 285.0394 | -3.72 | 285.0387/243.0287/241.0482/199.0387/175.0386/151.0028/133.0283/107.0140 | [M−H]− | Luteolin |
| 18 | 3.59 | C_16_H_12_O_7_ | 315.0501 | -2.94 | 301.1266/300.0272/272.0266/255.0071/243.0253/227.0311/136.9862 | [M−H]− | Isorhamnetin |
| 19 | 3.91 | C_15_H_10_O_5_ | 269.0443 | -4.63 | 269.0426//201.0543/181.0585/151.0036/149.0223/117.0316/107.0123 | [M−H]− | Apigenin |
| **Sesquiterpenes** | | | | | | | |
| 20 | 2.77 | C_15_H_20_O_5_ | 281.1383 | -0.18 | 265.6962/ 246.1944/ 217.1184/ 199.1083 | [M+H]+ | Psilostachyin |
| 21 | 2.83 | C_15_H_20_O_5_ | 281.1383 | -0.18 | 281.1347/ 264.1080/ 253.1020/233.1110/ 218.1224 | [M+H]+ | Vulgarolide |
| 22 | 2.92 | C_15_H_21_O_4_ | 265.1432 | -0.89 | 265.1432/ 247.1270/ 229.1200/ 211.1074 | [M+H-H_2_O]+ | Artemisinin |
|  | 3.22 | C_15_H_22_O_5_ | 281.1384 | -3.73 | 237.1476/219.1386 | [M−H]− | Artemisinin |
| 23 | 2.95 | C_15_H_20_O_4_ | 265.1432 | -0.89 | 247.1325/229.1200/201.1223/173.1297/131.0843 | [M+H]+ | Vulgarin |
| 24 | 2.98 | C_15_H_18_O_4_ | 263.1276 | -0.71 | 245.1168/233.1110/ 227.1061/ 199.1083/171.1161/91.0512/72.2907 | [M+H]+ | Artemisin |
| 25 | 3.29 | C_15_H_22_O_4_ | 267.1588 | -1.07 | 231.1376/203.1416 | [M+H]+ | Artemin |
| 26 | 3.61 | C_15_H_22_O_2_ | 235.1690 | -1.09 | 217.1542/199.1475 | [M+H]+ | Artemisinic acid |
| 27 | 4.06 | C_15_H_20_O_3_ | 249.1484 | -0.49 | 231.1376/203.1416/185.1283/173.0932/157.0976/145.0969/131.0843/117.0681/111.0788/105.0687 | [M+H]+ | Arteannuin B |
| 28 | 4.14 | C_15_H_24_O_3_ | 251.1645 | -3.06 | 205.5379/191.6805 | [M−H]− | α-Epoxy dihydroartemisinic |
| 29 | 4.3 | C_15_H_18_O_3_ | 247.1328 | -0.29 | 229.1200/201.1223/173.0932/168.0906/159.0798/153.0673/145.0969/135.0781/131.0843 | [M+H]+ | Santonin |
| 30 | 4.34 | C_15_H_20_O_2_ | 233.1532 | -1.74 | 215.1427/187.1465/159.1149/145.0969/131.0843/119.0831/105.0687/95.0844/81.0690 | [M+H]+ | Costunolide |
| 31 | 5.38 | C_15_H_24_O_2_ | 235.1703 | -0.23 | 217.1567/194.9874 | [M−H]− | Dihydroartemisinic acid |
| **Fatty acids** | | | | | | | |
| 32 | 5.13 | C_18_H_30_O_2_ | 279.2307 | 7.68 | 243.2068/235.1674/219.1751/137.1321/123.0798/109.0641/95.0844/81.0690/67.0540 | [M+H]+ | α-linolenic acid |

Identified secondary metabolites in A. vulgaris aqueous extract using UHPLC-QTOF-MS/MS analysis in both negative and positive ionization modes. RT: retention time; m/z: mass-to-charge ratio; ppm: parts per million. Fragment ions are presented as detected MS/MS peaks. Identification was based on molecular formula, fragmentation pattern, and comparison with reported literature.

**Table S2: Molecular docking of compounds exhibiting the strongest interactions against apoptotic and autophagic targeted proteins.**

| **Protein** | **Compound** | **2D** | **3D** | **binding energy (kcal/mol)** | **Amino acid residue interaction** | **Bonding type** |
| --- | --- | --- | --- | --- | --- | --- |
| **Apoptosis proteins** | | | | | |  |
| BCL2 | Co-crystallized ligand |  |  | -10.5 | ALA A:100 ALA A:149  ASN A:143 GLN A:99 GLY A:145 LEU A:137 MET A:115  PHE A:112  TYR A:103  TYR A:108 | Alkyl bond  π -Alkyl bond  Conventional H- bond  Conventional H- bond Conventional H- bond  π -Alkyl bond  π -Alkyl bond  π -sigma  Alkyl bond  Carbon H- bond |
|  | *Aesculin |  |  | -6.4 | *ALA A:149  ARG A:146 *ASN A:143 | Conventional H- bond & π -Alkyl bond  π -Alkyl bond  Conventional H- bond |
|  | *Coumaroylquinic acid |  |  | -7.0 | *ALA A:149  ARG A:146 PHE A:104 PHE A:112 *TYR A:108 | π -Alkyl bond  Conventional H- bond  π–π T-shaped  π-Doner H- bond  Conventional H- bond & π–π T-shaped |
|  | *Luteolin |  |  | -6.3 | *ALA A:100 *GLY A:145  TRP A:144 *TYR A:103 TYR A: 202 | π -Alkyl bond  Conventional H- bond  Conventional H- bond  π–π Stacked  π–π Stacked |
| P53 | Co-crystallized ligand |  |  | -7.4 | CYS A:220  PRO A:152  LEU A:145  PRO A:151  PRO A:223  VAL A:147 | Carbon H- bond & π-doner H-bond  π- Alkyl  π- Alkyl  π- Alkyl  π- Alkyl |
|  | *Aesculin |  |  | -7.4 | *CYS A:220  *LEU A:145  *PRO A: 151  *PRO A:223  THR A:150  THR A:230  THR A:231  *VAL A:147 | Carbon H- bond & π- Alkyl  Conventional H-bond  π- Alkyl  π- Alkyl  Conventional H-bond  Conventional H-bond  Conventional H-bond & carbon H-bond  π- Alkyl |
|  | *Chlorogenic acid |  |  | -7.3 | GLY A:154  *LEU A:145  *PRO A:151  *PRO A:152  PRO A:153  PRO A:222  *PRO A:223  THR A:155  THR A:230 | Conventional H-bond & unfavorable D-D  Conventional H-bond  Carbon H-bond  Conventional H-bond  π-Sigma  π- Alkyl  Carbon H-bond  Conventional H-bond  unfavorable D-D |
|  | *Coumaroylquinic acid |  |  | -7.3 | ASP A:228  GLU A:221  PRO A:219  *PRO A:223  THR A:231  *VAL A:147 | Conventional H-bond  Carbon H-bond &  Unfavorable D-D  Conventional H-bond  π- Alkyl  Conventional H-bond  π- Alkyl |
| **Autophagy proteins** | | | | | |  |
| LC3C | Co-crystallized ligand |  |  | -3.8 | ARG A:66  LEU A:49  LYS A:45 | Conventional H-bond & Salt bridge  Conventional H-bond  Conventional H-bond & Salt bridge |
|  | *Chlorogenic acid |  |  | -4.7 | ILE A:62  *LEU A:49 *LYS A:45  LYS A:47  PRO A:51  SER A:65 | π- Sigma  Conventional H-bond  Conventional H-bond  Conventional H-bond  Carbon H-bond  Conventional H-bond |
|  | *Coumaroylquinic acid |  |  | -4.7 | ILE A:62  *LEU A:49 *LYS A:45  PRO A:51 | π- Sigma  Conventional H-bond  Conventional H-bond  π-Alkyl |
|  | *Umbelliferone |  |  | -4.8 | ILE A:62  *LEU A:49  *LYS A:45  PHE A:48 | π- Sigma & π- Alkyl  Conventional H-bond  Conventional H-bond  π- π- Stacked |
| MTOR | Co-crystallized ligand |  |  | -5.9 | ARG A:1628  ARG A:1749  LYS A:1655 LYS A:1745  LYS A:1788 LYS A:1662 LYS A:1702 LYS A:1706 LYS A:1753 SER A:1658 TYR A:1698 | Attractive charge& salt bridge  Attractive charge& salt bridge  Conventional H- bond  Attractive charge& salt bridge  Conventional H-bond  Conventional H-bond  Conventional H-bond  Conventional H-bond  Conventional H-bond  Conventional H-bond  Conventional H-bond & Carbon H-bond |
|  | *Aesculin |  |  | -5.1 | *LYS A:1702 *LYS A:1706  *LYS A:1753 *SER A:1658 *TYR A:1698 TRP A:1786. *LYS A:1788, *LYS A:1662 | Conventional H- bond  Conventional H- bond &unfavorable D-D  Conventional H- bond  Conventional H- bond  Conventional H- bond  Conventional H- bond  π -Cation  unfavorable D-D |
|  | *Artemisinin |  |  | -5.4 | *ARG A:1749  *LYS A:1788 *TYR A:1698 | Conventional H- bond  & Carbon H-bond  Conventional H- bond  Conventional H- bond |
|  | *Rutin |  |  | -6 | *ARG A:1749  *LYS A:1662  *LYS A:1702 *LYS A:1788 *SER A:1658 *TYR A:1698 | Conventional H- bond &unfavorable D-D & Cabon H- bond  Conventional H- bond & π -Cation  Conventional H- bond  Conventional H- bond  Conventional H- bond  Conventional H- bond |

Molecular docking results of selected compounds showing the strongest binding interactions with apoptosis- (BCL2, P53) and autophagy-related (LC3C, mTOR) proteins. Binding energy is expressed in kcal/mol. Amino acid residues involved in ligand–protein interactions and types of bonding (e.g., hydrogen bonding, π-alkyl, π–π interactions) are presented. Co-crystallized ligands were used as reference standards.
