# Supplementary material for: Synergistic anti-cancer activity of Artemisia vulgaris L. aqueous extract with cisplatin against A549 lung cancer cell line
Source: BMC Complement Med Ther. 2026 Aug 11;26:242. doi: 10.1186/s12906-026-05481-5 (PMC13459383; doi:10.1186/s12906-026-05481-5)
Supplement: Supplementary file 3 [file 12906_2026_5481_MOESM3_ESM.docx]

**Table S3: The interactions between compounds and Apoptosis and Autophagy proteins**

|  |  |  |  | **Apoptosis** | | **Autophagy** | |
| --- | --- | --- | --- | --- | --- | --- | --- |
| **Class** | **Name** | **Pubchem id** | **Structure** | **BCL2** | **P53** | **LC3C** | **mtor** |
|  | **Co-crystallized ligand** |  |  | **-10.5** | **-7.4** | **-3.8** | **-5.9** |
| Phenolic acids | Quinic acid | 6508 |  | -4.6 | -5.7 | -4.0 | -3.9 |
|  | Coumaroylquinic acid | 6441280 |  | -6.3 | -7.3 | -4.7 | -4.7 |
|  | Chlorogenic acid | 1794427 |  | -6.5 | -7.3 | -4.7 | -4.9 |
| Coumarins | Aesculetin | 5281416 |  | -5.4 | -5.9 | -5.0 | -4.3 |
|  | Aesculin | 5281417 |  | -6.4 | **-7.4** | -5.4 | -5.1 |
|  | Umbelliferone | 5281426 |  | -5.8 | -5.8 | -4.8 | -4.0 |
| Flavonoids | Apigenin | 5280443 |  | -7.1 | **-7.4** | -5.3 | -4.7 |
|  | Luteolin | 5280445 |  | -7.0 | **-7.8** | -5.2 | -4.9 |
|  | Rutin | 6728944 |  | -6.4 | **-8.2** | **-3.2** | **-6.0** |
| sesquiterpene | Vulgarin | 94253 |  | -7.0 | -5.6 | -5.3 | -4.6 |
|  | Santonin | 221071 |  | -7.1 | -6 | -5.6 | -5.0 |
|  | Costunolide | 5281437 |  | -7.3 | -5.7 | -5.3 | -4.6 |
|  | Artemisinin | 68827 |  | -6.9 | -6 | -5.6 | -5.4 |

Comparative binding energies (kcal/mol) of selected phytoconstituents against apoptosis (BCL2, P53) and autophagy (LC3C, mTOR) target proteins. Compounds are classified according to their chemical classes. Binding energies approaching that of the co-crystallized ligand suggest stronger predicted binding affinity.

**Table S4: Molecular docking of the 13 compounds against BCL2 apoptotic protein**

| **Compound Name** | **2D** | **3D** |
| --- | --- | --- |
| Aesculetin |  |  |
| Aesculin |  |  |
| Apiginin |  |  |
| Artemisinin |  |  |
| Chloroginic acid |  |  |
| Costunolide |  |  |
| Coumaroylquinic acid |  |  |
| Luteolin |  |  |
| Prepared legand |  |  |
| Quinic acid |  |  |
| Rutin |  |  |
| Santonin |  |  |
| Umbelliferone |  |  |
| Vulgarin |  |  |

2D and 3D binding interactions of selected compounds docked against BCL2 apoptotic protein. The prepared ligand represents the co-crystallized reference ligand used for comparison. Visualizations illustrate binding orientation and key interaction sites within the active pocket.

**Table S5: Molecular docking of the 13 compounds against P53 apoptotic protein**

| **Compound name** | **2D** | **3D** |
| --- | --- | --- |
| Aesculetin |  |  |
| Aesculin |  |  |
| Apigenin |  |  |
| Artemisinin |  |  |
| Chlorogenic acid |  |  |
| Costunolide |  |  |
| Coumaroylquinic acid |  |  |
| Luteolin |  |  |
| Prepared ligand |  |  |
| Quinic acid |  |  |
| Rutin |  |  |
| Santonin |  |  |
| Umbelliferone |  |  |
| Vulgarin |  |  |

2D and 3D molecular docking interactions of selected compounds with P53 apoptotic protein. The prepared ligand corresponds to the co-crystallized reference ligand. Interaction diagrams highlight key residues involved in ligand binding.

**Table S6: Molecular docking of the 13 compounds against LC3C autophagic protein**

| **Compound name** | **2D** | **3D** |
| --- | --- | --- |
| Aesculetin |  |  |
| Aesculin |  |  |
| Apigenin |  |  |
| Artemisinin |  |  |
| Chlorogenic acid |  |  |
| Costunolide |  |  |
| Coumaroylquinic acid |  |  |
| Luteolin |  |  |
| Prepared ligand |  |  |
| Quinic acid |  |  |
| Rutin |  |  |
| Santonin |  |  |
| Umbelliferone |  |  |
| Vulgarin |  |  |

2D and 3D docking interactions of selected compounds with LC3C autophagy-related protein. The prepared ligand represents the reference ligand. Binding poses and interactions with active site residues are illustrated.

**Table S7: Molecular docking of the 13 compounds against mTOR autophagic protein**

| **Compound name** | **2D** | **3D** |
| --- | --- | --- |
| Aesculetin |  |  |
| Aesculin |  |  |
| Apigenin |  |  |
| Artemisinin |  |  |
| Chlorogenic acid |  |  |
| Costunolide |  |  |
| Coumaroylquinic acid |  |  |
| Luteolin |  |  |
| Prepared ligand |  |  |
| Quinic acid |  |  |
| Rutin |  |  |
| Santonin |  |  |
| Umbelliferone |  |  |
| Vulgarin |  |  |

2D and 3D molecular docking interactions of selected compounds with mTOR autophagy-related protein. The prepared ligand serves as the reference ligand. Key interactions and binding orientations within the active site are presented.

**Table S8:** **Alignment of co-crystallized ligand with the docked ligand pose (yellow)**

| **BCL-2** | **P53** |
| --- | --- |
|  |  |
| Rmsd: 0.724 | Rmsd: 1.361 |
| **LC3C** | **mTOR** |
|  |  |
| Rmsd: 1.901 | Rmsd: 2.690 |
